# Analysis of the Anti-Tumour Effect of Xuefu Zhuyu Decoction Based on Network Pharmacology and Experimental Verification in *Drosophila*

**DOI:** 10.3389/fphar.2022.922457

**Published:** 2022-07-12

**Authors:** Sitong Wang, Chenxi Wu, Yinghong Li, Bin Ye, Shuai Wang, Guowang Li, Jiawei Wu, Shengnan Liu, Menglong Zhang, Yongsen Jia, Huijuan Cao, Chunhua Jiang, Fanwu Wu

**Affiliations:** ^1^ Hebei Key Laboratory of Integrated Traditional Chinese and Western Medicine for Diabetes and Its Complications, College of Traditional Chinese Medicine, North China University of Science and Technology, Tangshan, China; ^2^ School of Traditional Chinese Medicine, Beijing University of Chinese Medicine, Beijing, China

**Keywords:** traditional Chinese medicine, Xuefu Zhuyu decoction, anti-tumour, network pharmacology, *drosophila*, cancer treatment

## Abstract

**Background:** Tumours are among the most lethal diseases that heavily endanger human health globally. Xuefu Zhuyu Decoction (XFZYD) is a prescription used to treat blood-activating stasis. Although XFZYD has been shown to suppress migration and invasion of tumour cells, the active ingredients, potential targets, and underlying mechanism remain largely elusive.

**Purpose:** To identify the prospective ingredients and major targets of XFZYD against tumours, and evaluate the efficacy and potential molecular mechanisms of XFZYD extract on tumour growth and invasion.

**Methods:** We predicted that XFZYD might act on 80 targets through 128 active components using the network pharmacology analysis method. In addition, we prepared an XFZYD aqueous extract and employed the Ras^V12^/*lgl* ^*−/−*^-induced *Drosophila* tumour model to carry out experimental verification.

**Results:** XFZYD did not exhibit any side effects on development, viability, and fertility. Furthermore, XFZYD significantly impeded tumour size and invasion at moderate concentrations and suppressed the increased phosphorylation of JNK but strongly enhanced the expression of Caspase 3 in the Ras^V12^/*lgl* ^*−/−*^ model. Finally, the mRNA level of the transcription complex AP-1 component *c-FOS* was remarkably reduced. In contrast, the transcription of three pro-apoptotic genes was significantly increased when XFZYD was used to treat the tumour model.

**Conclusion:** The study findings suggest that XFZYD may promote tumour cell apoptosis by activating caspase signalling to control primary growth and hinder tumour cell invasion by suppressing JNK/AP-1 signalling activity, thus providing a potential therapeutic strategy for XFZYD in the clinical treatment of cancer and other related diseases.

## Introduction

Cancers can be fatal diseases with high morbidity and mortality, posing a serious threat to global human health. According to Global Cancer Observatory, an estimated 19.3 million new cancer cases, and approximately 10.0 million cancer deaths occurred worldwide in 2020 (Sung et al., 2021). The occurrence and development of cancer-associated tumours are induced and controlled by many complicated factors, including cell proliferation, apoptosis, angiogenesis, autophagy, oxidative stress, immunity, and the microenvironment (Gonzalez et al., 2018; Aggarwal et al., 2019; Lugano et al., 2020).

Traditional Chinese Medicine (TCM) plays a role in the treatment of tumours through multiple factors, pathways, and targets (Xiang et al., 2019; Wang et al., 2021). Reportedly, TCM can inhibit tumour metastasis by stimulating apoptosis (Ge et al., 2015; Wu et al., 2018). For instance, Hu et al. (2007) showed that *Salvia miltiorrhiza* and *Paeoniae radix* extract induce apoptosis in a manner associated with Bcl-2 and the activation of Caspases 3 and 9 in HepG2 cells. Moreover, an extract from *Astragalus membranaceus* inhibits breast cancer cell growth through the PI3K/Akt/mTOR pathway (Zhou et al., 2018). Meanwhile, the Jiedu Sangen decoction inhibits colorectal cancer cell migration and invasion through the Hippo signalling pathway (Yuan et al., 2019).

Xuefu Zhuyu decoction (XFZYD) has been widely researched and applied clinically for the treatment of blood stasis syndrome (Leem et al., 2019; Tao et al., 2019), which has been described in ‘Yi Lin Gai Cuo’ (Corrections of Errors Among Physicians) by Wang Qingren in 1830s (Zhou et al., 2014). Blood stasis, characterised by blood flow retardation and stagnation (Goto, 2014; He et al., 2016), is an essential factor for tumourigenesis in TCM theory (Liu et al., 2007; Qian and Wang, 2009). Our previous studies showed that XFZYD inhibits cell migration and ocular tumour invasion by inhibiting epithelial-stromal transformation (EMT) (Wang et al., 2020). However, the components and molecular mechanism underlying the capacity of XFZYD to inhibit tumour metastasis have not been fully elucidated; hence, further studies are required.

Network pharmacology focuses on exploring the active compounds in drugs and their mechanisms of action. It is also widely applied to analyse the compatibility regularity of drug pairs and formulas in Chinese medicine research (Shao, 2021). With multiple genetic tools, for instance*,* UAS/GAL4, FLP/FRT, mosaic analysis with a repressible cell marker (MARCM) system (Zhang and Xue, 2012), and the conserved tumourigenesis machinery, *Drosophila melanogaster* has become an excellent model organism for probing tumour programs and drug targets *in vivo* (Rubin and Lewis, 2000; St Johnston, 2002; Gonzalez, 2013)*.* Therefore, in the current study, we employ network pharmacology analysis, as well as the oncogenic Ras (Ras^V12^) cooperated with *lgl* ^*4*^ homozygous mutant (Ras^V12^/*lgl* ^*−/−*^) mosaic clone-induced fly invasive tumour model (Pagliarini and Xu, 2003), to explore the prospective ingredients and major targets of XFZYD against tumours, while also evaluating the efficacy, and potential molecular mechanisms, of XFZYD extract on tumour growth and invasion to provide potential therapeutic strategies for XFZYD in the clinical treatment of cancer and associated diseases.

## Material and Methods

### Collection of Active Ingredients in Xuefu Zhuyu Decoction

XFZYD is composed of *Angelica sinensis (Oliv.) Diels.* (Dang Gui), *and Rehmannia glutinosa Libosch.* (Sheng Dihuang), *Prunus persica (L.) Batsch* (Tao Ren), *Carthamus tinctorius L.* (Hong Hua), *Citrus aurantium L.* (Fuchao Zhiqiao), *Paeonia lactiflora Pall.* (Chi Shao), *Bupleurum chinense DC.* (Bei Chaihu), *Glycyrrhiza uralensis Fisch.* (Gan Cao), *Platycodon grandiflorum (Jacq.) A. DC.* (Jie Geng), *Ligusticum chuanxiong Hort.* (Chuan Xiong), and *Cyathula officinalis Kuan* (Chuan Niuxi) (Supplementary Figure S1). These drug names were used as search keywords in the Traditional Chinese Medicine database and analysis platform (TCMSP, http://tcmspw.com/tcmsp.php). All chemical composition information of the 11 drugs in XFZYD were collected, and further screening was performed based on two key evaluation indicators: oral bioavailability (OB ≥ 30%) and drug-like parameters (DL ≥ 0.18). In addition, domestic and foreign literature was consulted as a supplement, and the potentially effective active ingredients in XFZYD were obtained.

### Collection and Analysis of Xuefu Zhuyu Decoction and Anti-Tumour Related Targets

The targets of XFZYD bioactive components were also searched from the TCMSP databases. The target corresponded with the target protein and gene information correction in the UniProt database (https://www.uniprot.org/) under the ‘Homo sapiens’ condition and was converted into a unified gene name.

GeneCards (www.genecards.org/) was used to acquire the relevant anti-tumour genes using the term ‘anti-tumour’ to screen for candidate targets. Only “Homo sapiens” proteins were selected, and the screening conditions were set at scores ≥1.81.

### Construction of Drug-Ingredient-Disease-Target Network

Cytoscape 3.7.0 (https://cytoscape.org/) was used to build network relationship diagrams and analyse the results. The node size was determined by the degree value, which varied from high to large. The transparency of the node colour was determined by the degree of the high value to the deep colour.

### Construction of Protein–Protein Interaction Network

The above targets were uploaded to the “Multiple proteins” function of the STRING platform (http://string-db.org) to demonstrate the interaction between target proteins. The species was set as “*Homo sapiens*”, and the “Interaction Score” was set to “Medium Confidence-0.400”. CytoHubba (Chin et al., 2014), a Cytoscape plug-in, was used to construct the Protein–Protein Interaction (PPI) network. Node size and colour were determined by the degree value, which was high for large and deep colours.

### Gene Ontology Enrichment and Kyoto Encyclopaedia of Genes and Genomes Pathway Analysis

The DAVID database (https://david.ncifcrf.gov/) was used to perform the Gene Ontology (GO) and Kyoto Encyclopaedia of Genes and Genomes (KEGG) pathway enrichment analysis. Statistical significance was set at *p* < 0.05. The histogram was plotted using http://www.bioinformatics.com.cn, a free online platform for data analysis and visualisation.

### Xuefu Zhuyu Decoction Extract Preparation

Eleven Chinese herbs in XFZYD were purchased from HeBei Linyitang Pharmaceutical Co., Ltd. The amounts for one dose, batch number, and producing area are listed in Supplementary Table S1 and Supplementary Table S2, respectively. All materials were authenticated by Prof. Chunyu Tian at the College of Traditional Chinese Medicine, North China University of Science and Technology.

The traditional prescriptions were prepared by the aqueous extraction method as previously described (Wang et al., 2020). The XFZYD extract was stored at −80°C, freeze-dried with a lyophiliser, and reconstituted in ddH_2_O to obtain a final stock concentration of 780 g/L.

### Quality Control of Xuefu Zhuyu Decoction

A fingerprint spectrum was established by high performance liquid chromatography (HPLC) to control the quality of XFZYD. The analyses were performed using a Shimadzu LC-20A instrument. The chromatographic column was an Agilent Eclipse XDB-C18 (4.6 × 250 mm, 5 μm). The mobile phase flow rate was 1 ml/min, and the column temperature was maintained at 35 ± 1°C. Quercetin, kaempferol, isorhamnetin, luteolin, and baicalein were dissolved in methanol as controls to be detected by HPLC. Quercetin, kaempferol, isorhamnetin, and luteolin were eluted using a gradient system consisting of methanol (A) and 0.4% phosphoric acid solution (B) (A:B, 50:50). Baicalein was eluted using a gradient system consisting of methanol (C) and 0.2% phosphoric acid solution (D) (C:D, 62:38).

### *Drosophila* Strains and Genetics

Flies were kept on a cornmeal and agar medium at 25°C in a 12 h light/dark cycle incubator according to standard protocols unless indicated. *Drosophila* stock *w* ^*1118*^ (#3605) was obtained from the Bloomington *Drosophila* Stock Centre (BDSC). To produce fluorescently labelled invasive tumours in the eye discs, the following strains were previously described (Pagliarini and Xu, 2003; Ma et al., 2017), including *yw ey*-Flp; *tub*-GAL80 FRT40A; *act*>*y* ^+^>GAL4 *UAS*-GFP, *w*; *lgl* ^*4*^ FRT40A *UAS-*Ras^V12^/*Cyo*, *ey-*Flp *act>y* ^*+*^ *>*GAL4 *UAS-*GFP, and *UAS*-Puc.

For all fly crossing experiments, healthy, unmated female, and male parents were randomly assigned to different groups. Crosses were kept on the same conditions as described above. The fly food was prepared as follows: 50 g/L sucrose, 10 g/L agar, 60 g/L polenta, 30 g/L yeast, and 6 ml propionic acid. The above ingredients were boiled with 1 L of ddH_2_O to prepare the regular food. XFZYD extract was added directly to regular food from 780 g/L aqueous stock to final concentrations of 1.57, 3.13, 6.25, and 12.5 g/L. Group + represents regular food without XFZYD. Groups 1.57, 3.13, 6.25, and 12.5 represent regular food with XFZYD in final concentrations of 1.57, 3.13, 6.25, and 12.5 g/L, respectively.

### Food Intake

Early third-instar larvae were washed with phosphate-buffered saline (PBS) and starved for 2 h under adverse food conditions (0.8% agar in PBS). Then the larvae were transferred to fresh dye-added food (0.05% Brilliant Blue) for 20 min. After feeding, larvae were washed with PBS, dried on tissue paper, and homogenized in 100 μL of lysis buffer (PBS + 0.1% Triton X-100). After centrifugation, 2 μL of the supernatant was analysed with a spectrophotometer at 630 nm. A small amount of dye-containing food was weighed, processed as described above, and used as a standard to calculate the amount of ingested food. Triplicate measurements on each group were conducted (Cao et al., 2022).

### *Drosophila* Tumour Invasion Model

The crossing scheme for establishing the Ras^V12^/*lgl* ^*−/−*^-induced tumour invasion model is shown in Supplementary Figure S2. Tumour invasion, marked by green fluorescent protein (GFP), was observed, and recorded. For the XFZYD-treated groups, offspring with genotype Ras^V12^/*lgl* ^*−/−*^ were kept on the XFZYD-added medium from the egg stage to the third instar larval stage or pupal stage. Female flies with genotype *ey-*Flp *act>y* ^*+*^ *>*GAL4 *UAS-*GFP were crossed with male flies of strain *w* ^*1118*^ on regular food, and the progeny larvae with genotype *ey-*Flp *act>y* ^*+*^ *>*GAL4 *UAS-*GFP/+ were collected as the control group. Female flies with genotype *yw ey*-Flp; *tub*-GAL80 FRT40A; *act*>*y* ^+^>GAL4 *UAS*-GFP were crossed with male flies of strain *w*; *lgl* ^*4*^ FRT40A *UAS-*Ras^V12^/*Cyo*; *UAS*-Puc/TM6B.Tb on regular food, and the progeny larvae with genotype *yw ey*-Flp/+; *tub*-GAL80 FRT40A/*lgl* ^*4*^ FRT40A *UAS-*Ras^V12^; *act*>*y* ^+^>GAL4 *UAS*-GFP/*UAS*-Puc (Ras^V12^/*lgl* ^*−/−*^+Puc) were selected as the positive control.

### Developmental Assay

Thirty female and 20 male *w* ^*1118*^ flies were crossed, and eggs were laid in regular food or medium with XFZYD. Thirty healthy L1 larvae were collected 24 h after egg deposition (AED) and reared in 30 animals/tube with a new corresponding medium. The percentage of white pupae was estimated every 4 h to generate a developmental curve, and the percentage of emerging adults was recorded to assess viability (Wu Q. et al., 2021).

### Fertility Assay

Parent *w* ^*1118*^ flies were kept in regular food or medium with XFZYD at a concentration of 1.57 g/L, 3.13 g/L, 6.25 g/L, or 12.5 g/L. The offspring of each group were selected for analysis when they reached adulthood after eclosion. For the fertility assay, female F1 flies or male F1 flies from each group were successfully mated by healthy *w* ^*1118*^ males or virgin *w* ^*1118*^ females and maintained separately on a standard medium. F1 flies were classified as fertile or sterile according to the presence or absence of offspring (Li et al., 2015).

### Immunohistochemistry

The dissected larval tissues were fixed in 4% formaldehyde for 20 min at 20°C. After three washes with 0.3% (v/v) PBST, the tissues were stained with primary antibodies overnight at 4°C and then with secondary antibodies at RT for 4 h. The following antibodies were used: rabbit anti-pJNK (1:200, Calbiochem, #559309), rabbit anti-cleaved Caspase 3 (1:400, Cell Signalling Technology, #9661), and goat anti-Rabbit-Cyanine3 (Cy3) (1:1000, Life Technologies, #A10520). Vectashield mounting medium (Vector Laboratories, H-1500) with 4′, 6-diamidino-2-phenylindole (DAPI) was used for mounting. The fluorophores DAPI, GFP, and Cy3 were excited and visualised using an inverted fluorescent microscope (Olympus, IX51).

### Real-Time Quantitative PCR (qRT-PCR)

TRIzol (Invitrogen) was used to isolate total RNA from five 3^rd^ instar larvae, and qRT-PCR was performed as previously described (Wang et al., 2003). *Rp49* was used as the internal control.

Primers used are provided:

For *rp49* Sense: 5′-TAC​AGG​CCC​AAG​ATC​GTG​AA-3′

Antisense: 5′-TCT​CCT​TGC​GCT​TCT​TGG​A-3′

For *c-Fos* Sense: 5′-CCT​TCA​AAT​GGG​CAG​ACA​A-3′

Antisense: 5′-TGG​GAG​CGA​CGA​AAC​ACC-3′

For *rpr* Sense: 5′-AAA​GTC​CGG​CAA​ATA​TCG​CAA​G-3′

Antisense: 5′-TGT​TGT​GGC​TCT​GTG​TCC​TTG​ACT-3′

For *hid* Sense: 5′-TGC​GAA​ATA​CAC​GGG​TTC​A-3′

Antisense: 5′-CCA​ATA​TAC​ACC​CAG​TCC​CG-3′

For *grim* Sense: 5′-TCG​GAG​TTT​GGA​TGC​TGG​GAT​C-3′

Antisense: 5′-AGT​CAC​GTC​GTC​CTC​ATC​GTT​GT-3′

### Statistical Analysis

The data reported were verified using at least three independent experiments. Image-Pro Plus 6.0 was used to calculate tumour size in pixels. Results were presented as bar graphs or line charts performed using GraphPad Prism 8.0. For statistical significance verification of the experiment, one-way analysis of variance (ANOVA) with Bonferroni’s multiple comparison test or chi-squared test was applied. The centre values were taken as the mean, and error bars indicated standard deviation. Statistical significance was set at *p* < 0.05 and *p* ≥ 0.05 was considered insignificant.

## Results

### Anti-tumour Related Candidate Targets of Active Ingredients of Xuefu Zhuyu Decoction

Based on the TCMSP databases, 186 active ingredients of XFZYD were screened, including two compounds from *Angellica sinensis (Oliv) Diels.*, two from *Rehmannia glutinosa Libosch*, 17 from *Bupleurum chinense*, 23 from *Prunus persica (L.) Batsch*, 22 from *Carthamus tinctorius L.*, five from *Citrus aurantium L.*, 29 from *Paeonia lactiflora Pall.*, 17 from *Bupleurum chinense DC.*, 92 from *Glycyrrhiza uralensis Fisch.*, 7 from *Platycodon grandiflorum (Jacq.) A. DC.*, 7 from *Ligusticum chuanxiong Hort*, and 4 compounds from *Cyathula officinalis Kuan*.

Based on these active ingredients in XFZYD, 273 active ingredients-related targets were acquired via TCMSP, and 289 anti-tumour-related targets were obtained from GeneCards. UniProtKB was used to obtain the standard names of the predicted target proteins. After merging active ingredient and anti-tumour-related targets, 80 overlapping targets were identified as candidates (Supplementary Table S3). A total of 128 candidate ingredients of XFZYD with anti-tumour activity were obtained (Supplementary Table S4). Of these, β-sitosterol, stigmasterol, quercetin, kaempferol, campesterol, baicalein, luteolin, naringenin, and isorhamnetin were present in more than one herbal medicine.

### Drug-Ingredient-Disease-Target Network Analysis

**FIGURE 1:**
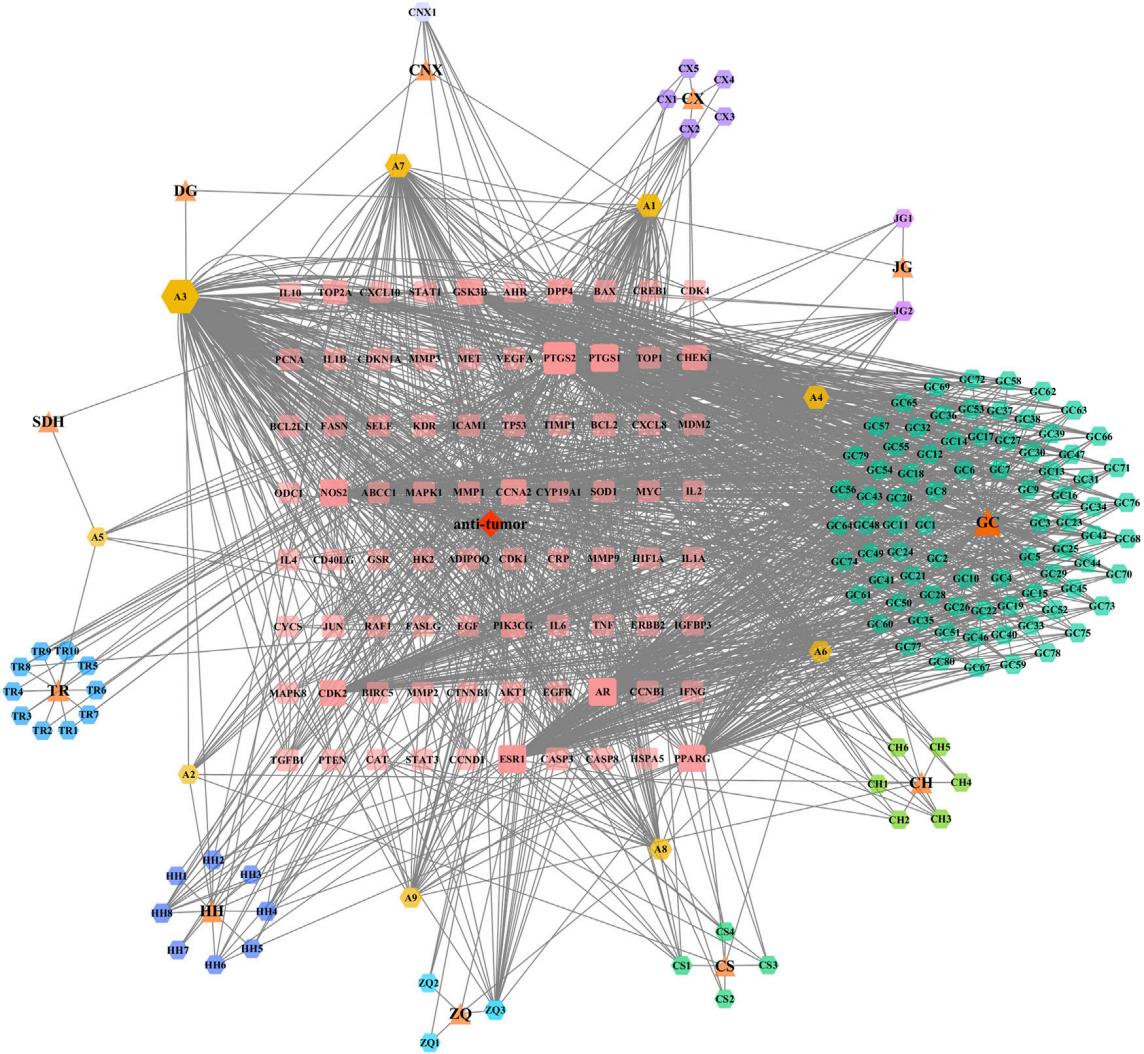
Bioinformatic analysis of XFZYD ingredients. XFZYD anti-tumour related targets network comprises 11 herbs, 128 ingredients, and 80 targets. The herbs are presented as triangles, the ingredients as hexagons, and the targets as rounded rectangles. Node size and transparency were determined by the degree and value depicted as differences in size and colour shade.

To further elucidate the key active ingredients and targets, we analysed the complex relationship between XFZYD, active ingredients, anti-tumour activity, and gene targets. The drug-ingredient-disease-target network of XFZYD was constructed using Cytoscape. As a result, 220 nodes and 1,572 edges were included in this network. The network showed 11 herbs, 128 active ingredients, and 80 key targets and their interrelationships with XFZYD (Figure 1).

### Protein–Protein Interaction Network Analysis

**FIGURE 2:**
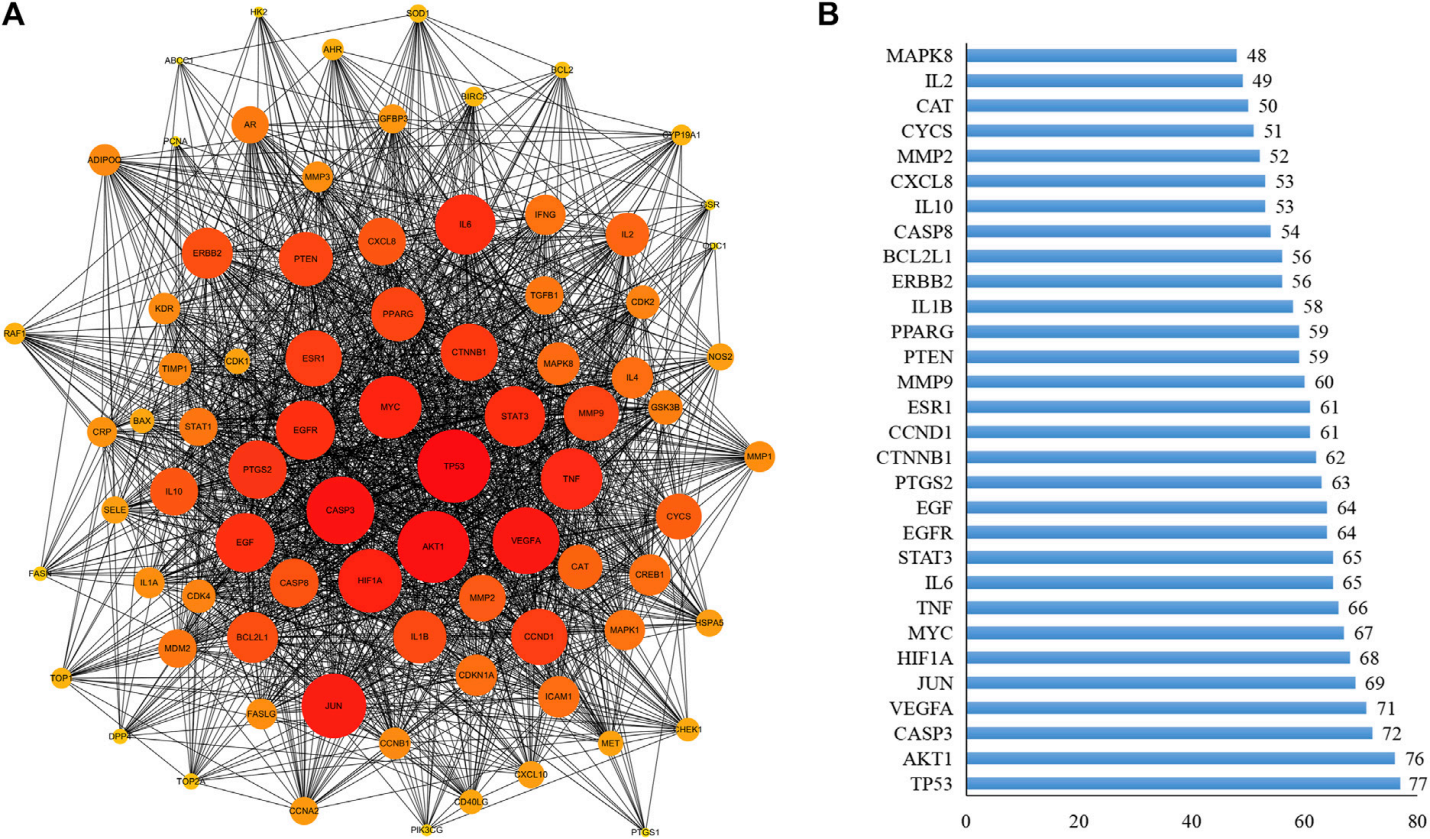
PPI analysis. **(A)** The PPI was constructed by STRING databases. Node size and depth of gene targets are proportional to the degrees; **(B)** PPI analysis for the top 30 targets.

A PPI network for the 80 key targets was constructed using Cytoscape. The network consists of 80 nodes and 1,692 edges in total (Figure 2A). The top 30 targets in this network ranked by the degree method were TP53, AKT1, CASP3, VEGFA, JUN, HIF1A, MYC, TNF, IL6, STAT3, EGFR, EGF, PTGS2, CTNNB1, CCND1, ESR1, MMP9, PTEN, PPARG, IL1B, ERBB2, BCL2L1, CASP8, IL10, CXCL8, MMP2, CYCS, CAT, IL2, and MAPK8 (Figure 2B), suggesting that these factors may be the central targets for XFZYD in the treatment of tumours.

### Gene Ontology Enrichment and Kyoto Encyclopaedia of Genes and Genomes Pathway Analysis of Xuefu Zhuyu Decoction

**FIGURE 3:**
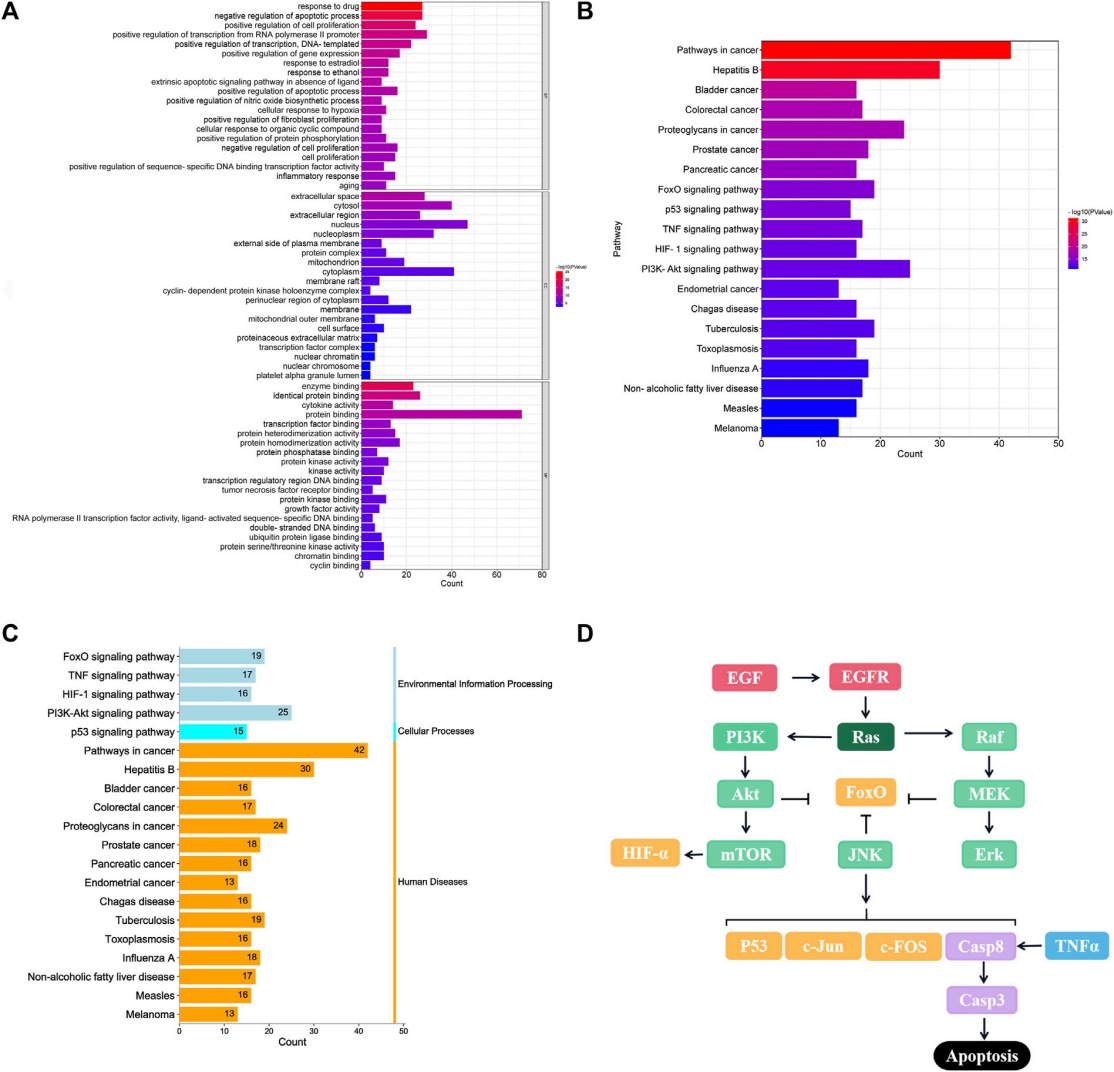
GO and KEGG enrichment analysis of 80 overlapped genes. **(A)** Top 20 GO enrichment analysis for the functional processes, molecular mechanism, and function; **(B)** Top 20 KEGG enrichment pathway; **(C)** Enrichment functional classification for top 20 KEGG pathways. **(D)** Crosstalk of key targets and pathways.

To investigate the cell functions primarily impacted by the candidate targets, we first performed GO enrichment analysis, which includes biological processes (BP), molecular functions (MF), and cellular components (CC) to elucidate the multiple biological functions of potential targets on the anti-tumour activity at a systemic level (Figure 3A). For BP, the potential targets of XFZYD were enriched in extrinsic apoptotic signalling pathway in the absence of ligand (GO:0097192), positive regulation of apoptotic process (GO:0043065), positive regulation of protein phosphorylation (GO:0001934), and negative regulation of cell proliferation (GO:0008285), etc. Regarding MFs, the targets were enriched in enzyme binding (GO:0019899), identical protein binding (GO:0042802), cytokine activity (GO:0005125), and protein binding (GO:0005515). Whereas the enriched CCs included extracellular space (GO:0005615), cytosol (GO:0005829), extracellular region (GO:0005576), and nucleus (GO:0005634).

To illustrate the crucial pathways affected by the potential targets, 80 targets were analysed for KEGG pathway enrichment. The top 20 pathways (*p* < 0.05) were screened (Figure 3B), including cancer (hsa05200), bladder cancer (hsa05219), colorectal cancer (hsa05210), proteoglycans in cancer (hsa05205), and prostate cancer (hsa05215) pathways. In addition, FOXO (hsa04068), TNF (hsa04668), p53 (hsa04115), HIF-1 (hsa04066) and PI3K-Akt signalling pathways (hsa04151) were recognised as the classical cancer-related signalling pathways (Figure 3C), that engage in complex crosstalk, based on previous experimental studies (Figure 3D).

### Xuefu Zhuyu Decoction Preparation and High Performance Liquid Chromatography Analysis

**FIGURE 4:**
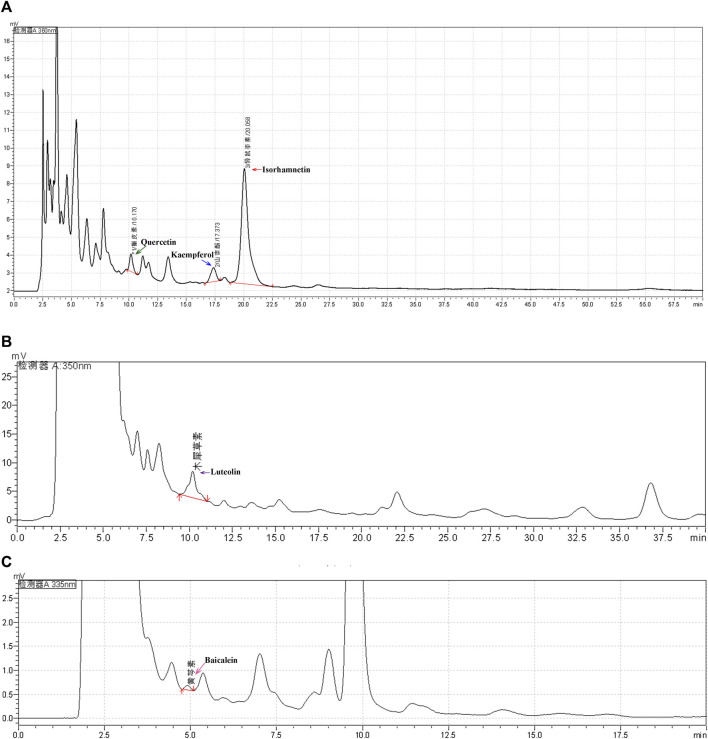
The HPLC chromatogram of XFZYD. HPLC analysis of five chemical constituents, quercetin (**a**, 360 nm), kaempferol [**(A)**, 360 nm], isorhamnetin [**(A)**, 360 nm], luteolin [**(B)**, 350 nm), and baicalein [**(C)**, 335 nm], in XFZYD. The tested peaks are indicated by green, blue, red, purple, and pink arrows.

To better verify the anti-tumour effect of XFZYD, we first prepared an XFZYD aqueous extract and assessed its quality. Five representative ingredients were selected as quality control standards. When the XFZYD HPLC chromatogram was compared with the standards (Supplementary Figure S3), we observed distinct peaks for quercetin, kaempferol, isorhamnetin, luteolin, and baicalein in the chemical fingerprints of XFZYD (Figures 4A–C). The contents of these five compounds in XFZYD, as determined by HPLC analysis, were 0.0026%, 0.0031%, 0.040%, 0.0016%, and 0.00035%, respectively. Collectively, these data indicate that XFZYD contains essential active ingredients and is appropriate for use in further *in vivo* verification studies.

### Effect of Xuefu Zhuyu Decoction on the Development, Viability, and Fertility of *Drosophila*

**FIGURE 5:**
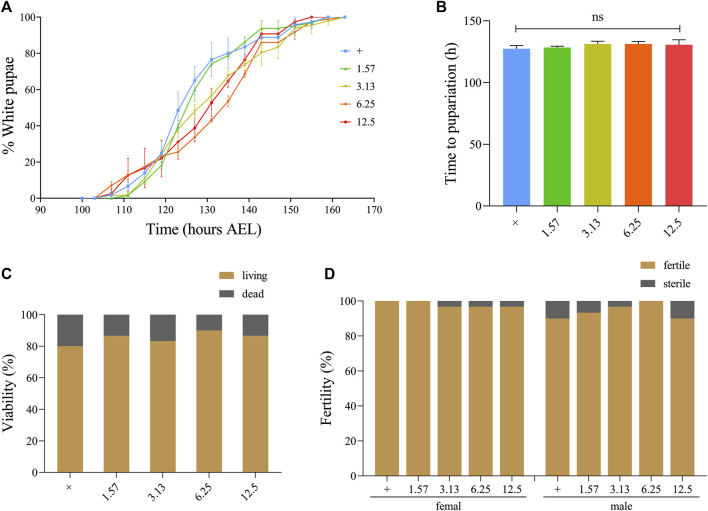
Effect of XFZYD on the development, viability, and fertility of *Drosophila*. Statistical analysis of the white pupae rate [**(A)**, *n* = 3] and time to pupariation [**(B)**, *n* = 3] of *w* ^*1118*^ larvae fed control food, or XFZYD-added food are shown. For time to pupariation statistics, a one-way ANOVA with Bonferroni’s multiple comparison test was applied, and ns indicates no significant difference. For the histogram of viability [**(C)**, *n* = 30] and fertility [**(D)**, *n* = 30] in *w* ^*1118*^ adults, the chi-squared test was applied (*n* = 30).

To determine whether XFZYD affects the normal growth, development, and reproductive capacity of living organisms, we selected the classic model organism *Drosophila melanogaster* to perform developmental and fertility assays. The white pupal curve and mean pupation time of *w* ^*1118*^ larvae, raised in fly mediums supplemented with different concentrations of XFZYD extract, were not affected compared to the control group (Figures 5A,B). There was no significant change in the viability or fertility of *Drosophila* adults between the control and XFZYD-added groups (Figures 5C,D). Hence, we concluded that XFZYD does not adversely affect development, viability, or fertility in *Drosophila*.

### Xuefu Zhuyu Decoction Suppresses Ras^V12^/*lgl* ^*−/−*^ Induced Tumour Growth and Invasion

**FIGURE 6:**
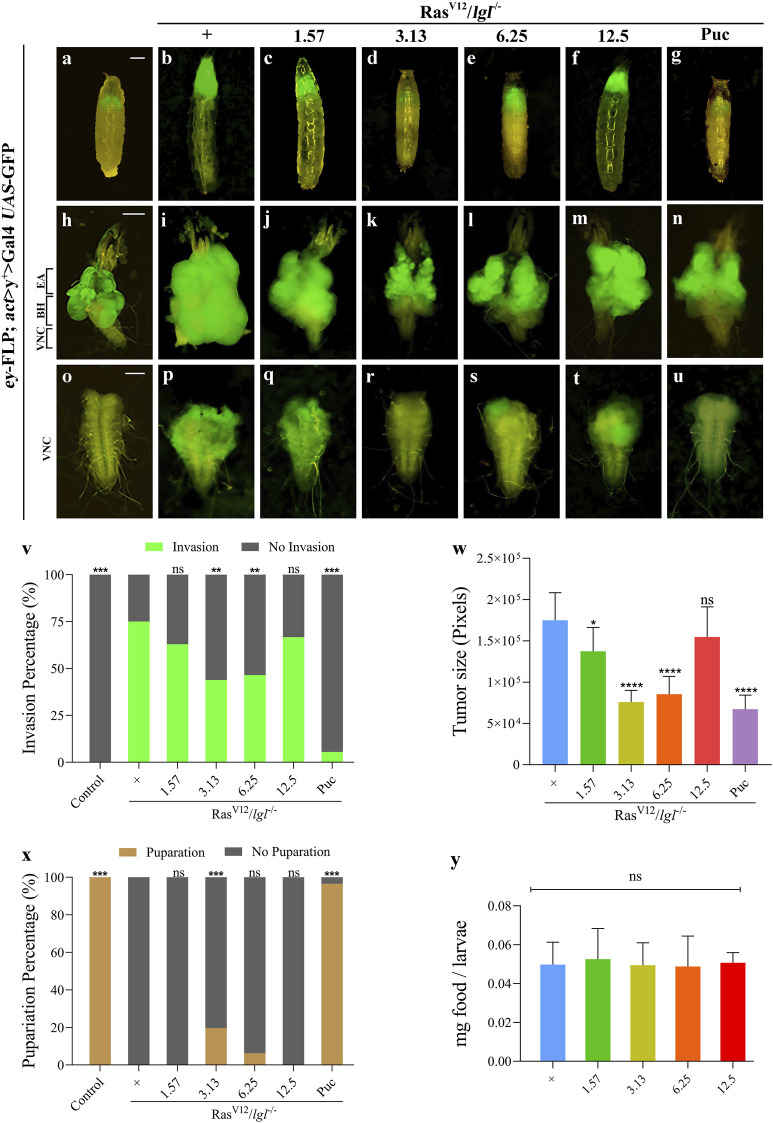
XFZYD inhibits tumour invasion in *Drosophila* VNC. Fluorescent images showing *Drosophila* larval whole body **(A–G)**, cephalic complexes **(H–N)**, and ventral nerve cords [VNC, **(O–U)**]; the anterior is up in all panels. Statistical analysis of the invasion percentage **(V)** is shown in figures **(O–U)** [**(O)**, 0%, *n* = 50; **(P)**, 75%, *n* = 44; **(Q)**, 63%, *n* = 46; **(R)** 43.9%, *n* = 57; **(S)**, 46.5%, *n* = 43; **(T)**, 66.7%, *n* = 45; **(U)**, 5.5%, *n* = 55]. The histogram of tumour size **(W)** is shown in figures **(I–N)**, and is measured in pixels by Image-Pro Plus 6.0 (*n* = 10). Stacked bar graph of the pupariation percentage **(X)** is shown in figures **(A–G)** [**(A)**, 100%, *n* = 50; **(B)**, 0%, *n* = 51; **(C)**, 0%, *n* = 52; **(D)** 19.67%, *n* = 61; **(E)**, 6.25%, *n* = 43; **(F)**, 0%, *n* = 45; **(G)**, 96.6%, *n* = 58]. **(Y)** Measurement of 20 min food intake by early third-instar larvae as calculated by blue food ingestion (6–8 larvae per pool, *n* = 3). For invasion and pupariation statistics, the chi-square test was applied. For tumour size and food intake statistics, one-way ANOVA with Bonferroni’s multiple comparison test was applied. Compared to the Ras^V12^/*lgl* ^*−/−*^ group, ****p < 0.0001, ***p < 0.001, **p < 0.01, *p < 0.05; ns, no significant difference. Scale bar: 500 µm **(A–G)**, 200 µm **(H–N)** and 100 µm **(O–U)**.

Considering the potential targets predicted by the network pharmacology analysis, we employed the well-established *eyeless* (*ey*)-Flp/MARCM system-mediated Ras^V12^/*lgl* ^*−/−*^ tumour invasion model at the *Drosophila* 3^rd^ instar larval stage to investigate the anti-tumour effects of XFZYD *in vivo*. In line with previous studies, ectopic expression of activated Ras (Ras^V12^) in *lgl* ^*4*^ homozygous mutant (*lgl* ^*−/−*^) mosaic clones triggered noticeable tumour-like overgrowth at the cephalic complexes, which consist of the brain hemispheres (BH) and the eye-antennal discs (EA), labelled with GFP (Figures 6B,I), and invasive metastasis to the ventral nerve cord (VNC) of the central nervous system (Figure 6P). For the control, neither tumour growth nor invasion was observed (Figures 6A,H,O). In addition, Ras^V12^/*lgl* ^*−/−*^-mediated tumour invasion caused an extended larval period, as well as interfered with the normal development of larvae into the pupal stage. Ultimately, the animals died as bloated 3^rd^ instar larvae (Figures 6B,X).

To further explore the inhibitory effect, we prepared fly food with the XFZYD aqueous extract at concentrations of 1.57 g/L, 3.13 g/L, 6.25 g/L, or 12.5 g/L and raised the Ras^V12^/*lgl* ^*−/−*^ flies. Compared with the model (Figures 6B,I,P), the XFZYD extract at a concentration of 3.13 g/L (Figures 6D,K,R) or 6.25 g/L (Figures 6E,L,S) largely suppressed *in-situ* growth and invasion rate of the tumour and 3.13 g/L XFZYD recovered the pupariation rate from 0 to 19.67% (*p <* 0.05) (Figures 6V–X). Furthermore, we observed that XFZYD at a concentration of 1.57 g/L (Figures 6C,J,Q) reduced the tumour size with no effect on the invasion rate and pupariation rate. Moreover, XFZYD at a concentration of 12.5 g/L did not cause any significant changes (Figures 6F,M,T). The tumorigenesis and pupariation were strongly rescued by the expression of Puckered (Puc) (Figures 6G,N,U–X), which served as a positive control (Wu C. et al., 2021). The effect of XFZYD on the invasion percentage and tumour size is not decreased in a dose-dependent manner. Thus, a concentration of 3.13 g/L of XFZYD aqueous extract significantly inhibited tumour growth and invasion and was selected to further explore the molecular mechanisms.

Next, we measured the level of food intake and observed a normal ingestion rate in both the XFZYD-added and the control groups (Figure 6Y), ruling out the possibility that changes in feeding rate may interfere with the nutritional status and tumour phenotypes.

### Xuefu Zhuyu Decoction Represses JNK Activity and Promotes Caspase-mediated Apoptosis

**FIGURE 7:**
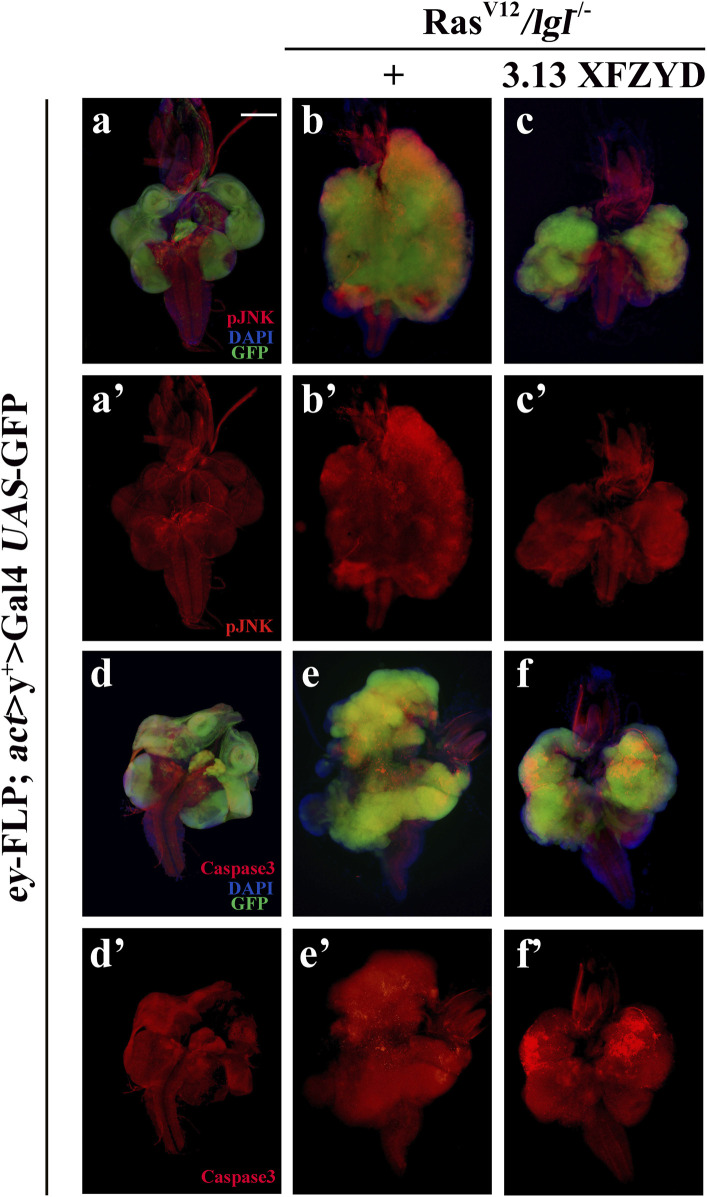
XFZYD alters pJNK and Caspase3 expression. Merged fluorescence micrographs of 3^rd^ instar larval cephalic complexes **(A–F)** are shown. The individual channels detect only pJNK [red, **(A’–C’)**] or only cleaved-Caspase3 signal [red, **(D’–F’)**]. Tumour cells are marked with a green fluorescent protein (GFP), and nuclei (DNA) are labelled with DAPI (blue). Scale bar: 200 µm **(A–F)**.

To understand the mechanism by which XFZYD inhibits Ras^V12^/*lgl* ^*−/−*^-induced tumour growth and invasion, we considered TNF/JNK and caspase signalling as putative central targets. First, these factors, including TNF, MAPK8 (also termed JNK1), MYC, JUN, and MMP1, ranked in the top 30 of the PPI analysis (Figure 2B), are well-known components of the classic TNF/JNK pathway in flies and mammals (Xia and Karin, 2004). Second, the Casp8/Casp3 cascade is the key initiator protease/effector caspase cascade that induces extrinsic apoptosis and is required to prevent tissue damage (Shalini et al., 2015). Third, studies have shown that the JNK pathway involved in tumour invasion (Igaki et al., 2006; Ma et al., 2017), and the activity of JNK visualised by pJNK staining, are significantly increased in Ras^V12^/*lgl* ^*−/−*^-induced tumours (Figures 7A,B, A’,B’). In support of this assumption, we observed that the Ras^V12^/*lgl* ^*−/−*^ promotion of JNK activity was notably inhibited by treatment with XFZYD extract (Figures 7C, C’). In addition, the slight activation of the caspase cascade by Ras^V12^/*lgl* ^*−/−*^ oncogenic cooperation was greatly enhanced by XFZYD, thus initiating massive apoptosis, and reducing tumour size (Figures 7D–F, D’–F’).

**FIGURE 8:**
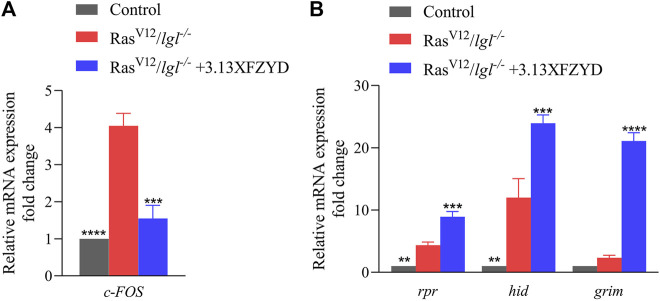
XFZYD regulates the transcription of *c-FOS*, rpr, hid, and grim. mRNA levels of *c-FOS* **(A)**, *rpr*, *hid*, and *grim* **(B)**, as measured by qRT-PCR (*n* = 3). Error bars represent the standard deviation. One-way ANOVA with Bonferroni’s multiple comparison test was used to compute *p-values*. ****p < 0.0001, ***p < 0.001, **p < 0.01.

Consistently, Ras^V12^/*lgl* ^*−/−*^ tumour in larva obviously increased the mRNA level of *c-FOS* that encodes one component of AP-1 (JNK downstream transcription complex) (Uhlirova and Bohmann, 2006), and the increased *c-FOS* transcription was notably impeded by XFZYD at the concentration of 3.13 g/L through a qRT-PCR assay (Figure 8A). Furthermore, XFZYD could strongly improve the transcription of three pro-apoptotic genes, *reaper* (*rpr*), *head involution defective* (*hid*), and *grim* (Figure 8B), which act upstream and initiate caspase-mediated apoptosis in *Drosophila* (Ge et al., 2012). Hence, these data suggest that XFZYD inhibits tumour growth and invasion by repressing JNK signalling and promoting caspase-mediated apoptosis.

## Discussion

We applied network pharmacology and drug target prediction to further explore the anti-tumour mechanism of XFZYD. Our study showed that XFZYD anti-tumour activity might act on 80 targets through 128 active components. The main active components were β-sitosterol, stigmasterol, quercetin, kaempferol, campesterol, baicalein, luteolin, naringenin, and isorhamnetin. The PPI network showed that CASP3, JUN, TNF, MMPs, and MAPK are the core hubs of XFZYD in tumour treatment. XFZYD has the advantages of multiple active ingredients and targets, which can possess various anti-tumour activities providing new opportunities for discovering targeted small molecules of “yet to be drugged” or “undruggable” proteins.

Over the last 20 years, *Drosophila melanogaster* has been considered a good model system for studying the genetic and molecular mechanisms of tumourigenesis and metastasis (Gerlach and Herranz, 2020). Additionally, studies have shown that feeding XFZYD has no side effects on normal development, viability, or fertility in *Drosophila.* Thus, the Ras^V12^/*lgl* ^*−/−*^-induced tumour invasion model was employed to investigate the anti-tumour effect of XFZYD. As shown in our previous study, XFZYD at a concentration of 0.0125 g/ml (12.5 g/L) elicits an obvious inhibitory effect on cell invasion (Wang et al., 2020). Thus, we selected the 1.57, 3.13, 6.25, and 12.5 g/L gradient concentrations for the Ras^V12^/*lgl* ^*−/−*^-induced tumour study based on the previous experiment. Collectively, 1.57 g/L, 3.13 g/L, and 6.25 g/L of XFZYD all reduced tumour size. Additionally, 3.13 g/L XFZYD strongly suppressed tumour invasion and rescued the pupariation rate in the Ras^V12^/*lgl* ^*−/−*^ tumour model.

Based on quantifications of daily *Drosophila* food intake (Deshpande et al., 2014), it is estimated that flies raised on media containing 1.57, 3.13, 6.25, and 12.5 g/L ingest about 0.4 mg/kg body weight of the drug per day, which is, respectively, comparable to the treatment dosage of 3.27, 6.52, 13.02 or 26.04 g per day for human patients. Actually, patients with coronary heart disease were treated with a single daily dose of XFZYD (78 g) in the clinic (Zhang et al., 2015). Thus, administering 6.52 g/day of XFZYD to humans (equivalent to 3.13 g/L for the fly) is far below the clinical medication dosage (78 g/day). Above all, the data may provide a key clue for the application of XFZYD in cancer clinical treatment.

Personalized treatment based on TCM theory plays a key role in cancer treatment and is consistent with the concept of precision medical treatment for cancer (Chen and Wang, 2012). TCM compounds and TCM syndromes are helpful for individualised therapy of cancer (Wang et al., 2018). Our study shows that different concentrations of XFZYD play active roles in inhibiting *Drosophila* tumour models caused by different gene mutations. The effect of XFZYD on the invasion percentage and tumour size is not decreased in a dose-dependent manner. Our findings suggest that the dosage of XFZYD should be determined according to the specific situation of the patients receiving the clinical treatment for tumour metastasis.

Our previous study showed that XFZYD inhibits tumour cell migration by regulating MMP1 expression (Wang et al., 2020). Moreover, the JNK-activated transcription complex (AP-1) regulates the expression of MMP1 (Uhlirova and Bohmann, 2006). To further elucidate the mechanisms underlying tumour invasion regulation by 3.13 g/L XFZYD, certain key bio targets were validated in this study. As JNK and effector caspases are linked during an invasion, tumours may further induce a metastatic cascade under apoptotic stress (Rudrapatna et al., 2013). We examined the expression of pJNK and Caspase3 and observed that they were upregulated in the Ras^V12^/*lgl* ^*−/−*^ tumour invasion model. However, the expression of pJNK decreased, while that of cleaved Caspase3 increased following XFZYD treatment. Furthermore, XFZYD decreased the expression of *c-FOS* and increased the mRNA levels of *rpr*, *hid*, and *grim* in the Ras^V12^/*lgl* ^*−/−*^ background, indicating that XFZYD may hinder tumour cell invasion by suppressing JNK/AP-1 signalling and promoting tumour cell apoptosis by activating caspase signalling.

## Conclusion

In summary, our work demonstrates that XFZYD displays anti-tumour activity at moderate concentrations. XFZYD impedes tumour invasion, presumably by inhibiting JNK signalling, and stimulating caspase-mediated apoptosis, thus, highlighting specific key drug targets and crucial strategies for cancer clinical treatment.

## Data Availability

The original contributions presented in the study are included in the article/Supplementary Material, further inquiries can be directed to the corresponding authors.

## Abbreviations

XFZYD, xuefu zhuyu decoction; TCM, traditional chinese medicine; EMT, epithelial-mesenchymal transition; MARCM, mosaic analysis with a repressible cell marker; TCMSP, traditional chinese medicine database and analysis platform; HPLC, high-performance liquid chromatography; PBS, phosphate-buffered saline; GFP, green fluorescent protein; AED, after egg deposition; qRT-PCR, real-time quantitative PCR; BP, biological processes; MF, molecular functions; CC, cellular components; BH, brain hemispheres; EA, eye-antennal discs.
