# Supplementary figures and images for: Analysis of the Anti-Tumour Effect of Xuefu Zhuyu Decoction Based on Network Pharmacology and Experimental Verification in Drosophila
Source: Front Pharmacol. 2022 Jul 12;13:922457. doi: 10.3389/fphar.2022.922457 (PMC9315317; doi:10.3389/fphar.2022.922457)

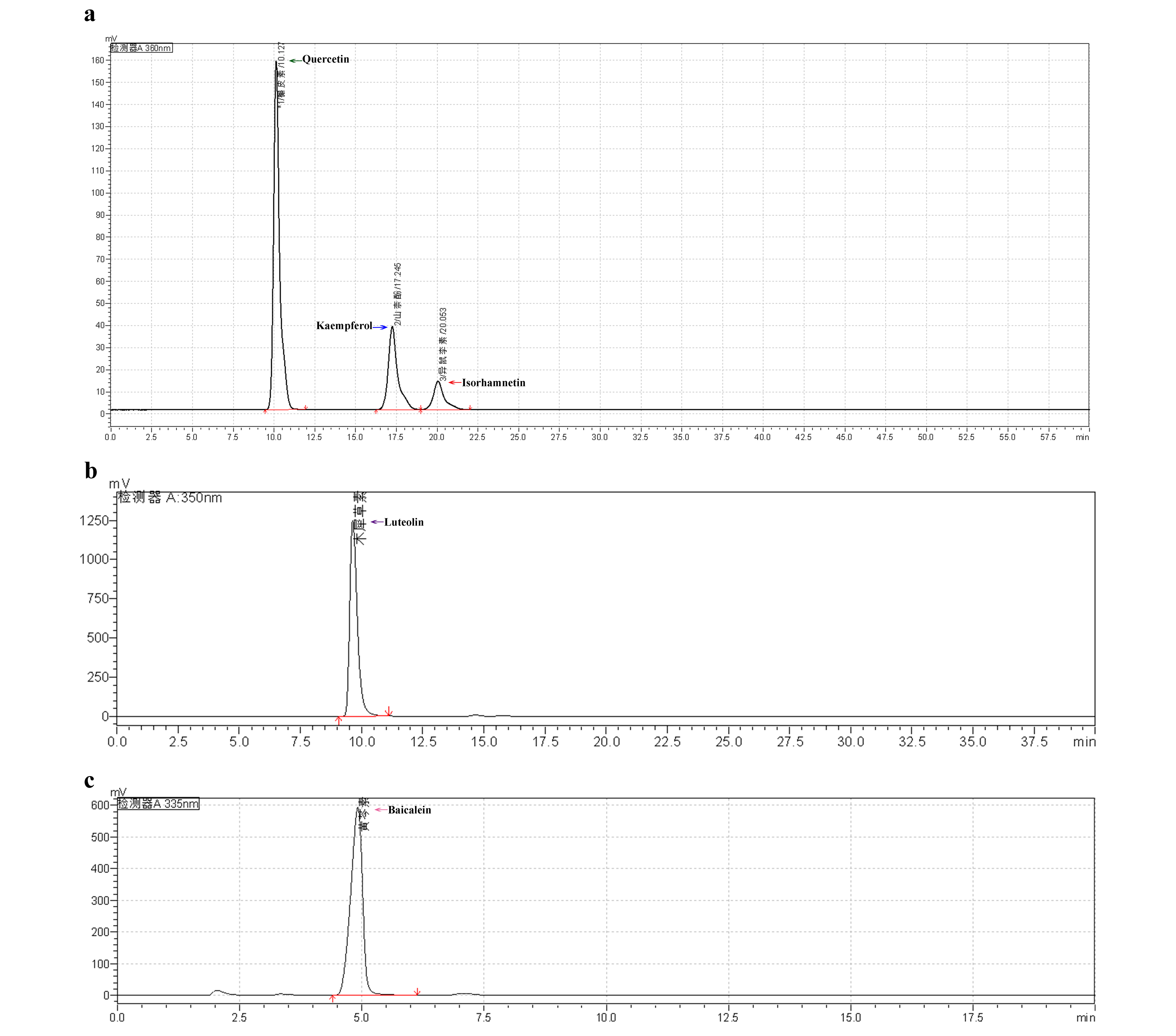

Supplement: Supplementary file 1 [file Image3.JPEG]

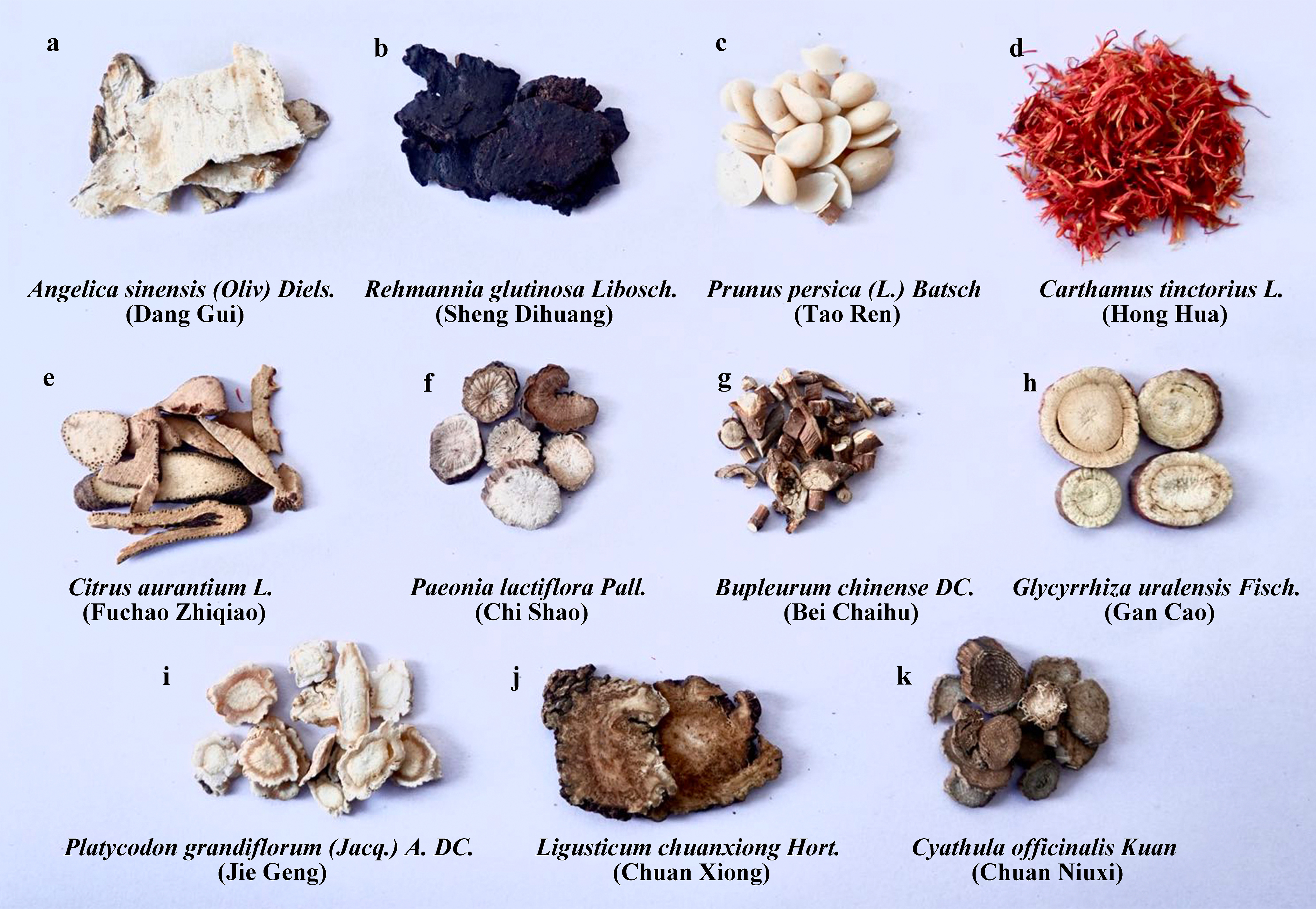

Supplement: Supplementary file 3 [file Image1.JPEG]

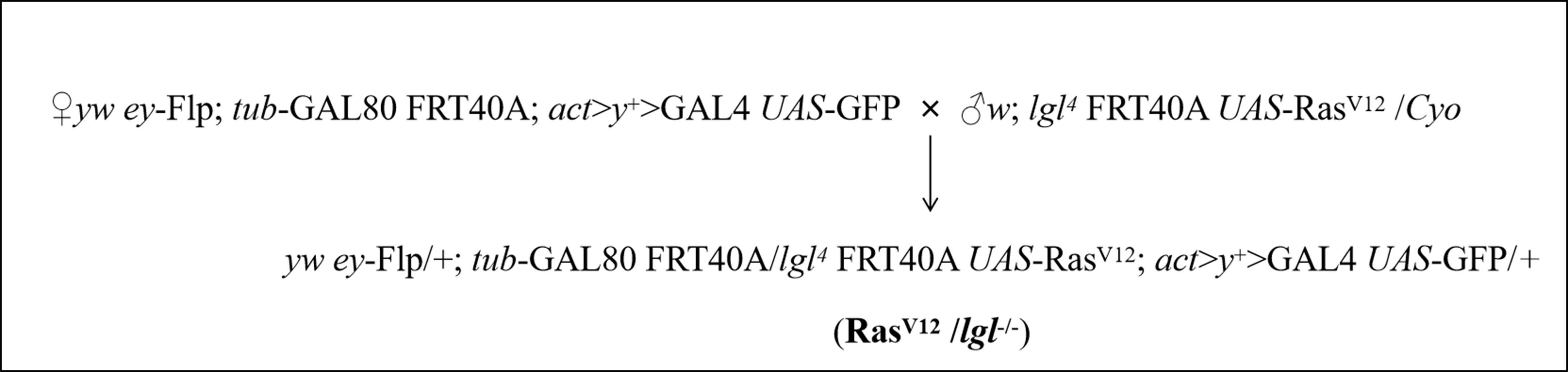

Supplement: Supplementary file 4 [file Image2.JPEG]
