# Supplementary material for: Analysis of the Anti-Tumour Effect of Xuefu Zhuyu Decoction Based on Network Pharmacology and Experimental Verification in Drosophila
Source: Front Pharmacol. 2022 Jul 12;13:922457. doi: 10.3389/fphar.2022.922457 (PMC9315317; doi:10.3389/fphar.2022.922457)
Supplement: Supplementary file 2 [file Table1.DOCX]

**Analysis of** **the** **anti-tumour effect of Xuefu Zhuyu decoction** **based on network pharmacology and** **experimental** **verification in *Drosophila***

**Sitong Wang^1,2†^, Chenxi Wu^1†^, Yinghong Li^1^, Bin Ye^1^, Shuai Wang^1^, Guowang Li^1^, Jiawei Wu^1^, Shengnan Liu^1^, Menglong Zhang^1^, Yongsen Jia^1^, Huijuan Cao^1^, Chunhua Jiang^1*^ and** **Fanwu Wu^1*^**

^1^ Hebei Key Laboratory of Integrated Traditional Chinese and Western Medicine for Diabetes and Its Complications, College of Traditional Chinese Medicine, North China University of Science and Technology, 21 Bohai Road, Tangshan 063210, China

^2^ School of Traditional Chinese Medicine, Beijing University of Chinese Medicine, Beijing 100029, China

^†^ These authors have contributed equally to this work and share first authorship.

^*^ **Correspondence**：

Chunhua Jiang, Fanwu Wu

[jiangchunhua@ncst.edu.cn](mailto:jiangchunhua@ncst.edu.cn), [ldwfw@sina.com](mailto:ldwfw@sina.com)

**Supplementary Tables**

**Wang *et al*., Table 1**

**Table 1. The formulation of XFZYD (one dose)**

| **Herb (Chinese name)** | **Medicinal Part** | **Amount in Application (g)** |
| --- | --- | --- |
| *Angelica sinensis* *(Oliv) Diels.* (Dang Gui) | Root | 9.0 |
| *Rehmannia glutinosa Libosch.* (Sheng Dihuang) | Root | 9.0 |
| *Prunus persica (L.) Batsch* (Tao Ren) | Seed | 12.0 |
| *Carthamus tinctorius L.* (Hong Hua) | Flower | 9.0 |
| *Citrus aurantium* *L.* (Fuchao Zhiqiao) | Fruit | 6.0 |
| *Paeonia lactiflora Pall.* (Chi Shao) | Root | 6.0 |
| *Bupleurum chinense DC.* (Bei Chaihu) | Root | 3.0 |
| *Glycyrrhiza uralensis* *Fisch.* (Gan Cao) | Root | 6.0 |
| *Platycodon grandiflorum* *(Jacq.) A. DC.* (Jie Geng) | Root | 4.5 |
| *Ligusticum chuanxiong Hort.* (Chuan Xiong) | Root | 4.5 |
| *Cyathula officinalis Kuan* (Chuan Niuxi) | Root | 9.0 |

**Wang *et al*., Table 2**

**Table 2. Batch number and producing area of drugs in XFZYD**

| **Herb (Local name)** | **Batch Number** | **Producing Area** |
| --- | --- | --- |
| *Angelica sinensis (Oliv) Diels.* (Dang Gui) | 20210120 | Gan Su |
| *Rehmannia glutinosa Libosch.* (Sheng Dihuang) | 20201204 | He Nan |
| *Prunus persica (L.) Batsch* (Tao Ren) | 20201120 | Liao Ning |
| *Carthamus tinctorius L.* (Hong Hua) | 20210120 | Xin Jiang |
| *Citrus aurantium L.* (Fuchao Zhiqiao) | 20201208 | Jiang Xi |
| *Paeonia lactiflora Pall.* (Chi Shao) | 088201002 | Hei Longjiang |
| *Bupleurum chinense DC.* (Bei Chaihu) | 050201201 | Shan Xi |
| *Glycyrrhiza uralensis Fisch.* (Gan Cao) | 20210308 | Xin Jiang |
| *Platycodon grandiflorum* *(Jacq.) A. DC.* (Jie Geng) | 191201 | He Bei |
| *Ligusticum chuanxiong Hort.* (Chuan Xiong) | 190401 | Si Chuan |
| *Cyathula officinalis* *Kuan* (Chuan Niuxi) | 20201222 | Si Chuan |

**Wang *et al*., Table 3**

**Table 3.** **Information on the anti-tumour targets of XFZYD**

| No. | Gene | Entrez Gene | No. | Gene | Entrez Gene | No. | Gene | Entrez Gene |
| --- | --- | --- | --- | --- | --- | --- | --- | --- |
| 1 | MMP2 | [4313](https://www.ncbi.nlm.nih.gov/gene/4313) | 28 | PIK3CG | [5294](https://www.ncbi.nlm.nih.gov/gene/5294) | 55 | ICAM1 | [3383](https://www.ncbi.nlm.nih.gov/gene/3383) |
| 2 | CXCL10 | [3627](https://www.ncbi.nlm.nih.gov/gene/3627) | 29 | MDM2 | [4193](https://www.ncbi.nlm.nih.gov/gene/4193) | 56 | MYC | [4609](https://www.ncbi.nlm.nih.gov/gene/4609) |
| 3 | GSR | [2936](https://www.ncbi.nlm.nih.gov/gene/2936) | 30 | CTNNB1 | [1499](https://www.ncbi.nlm.nih.gov/gene/1499) | 57 | JUN | [3725](https://www.ncbi.nlm.nih.gov/gene/3725) |
| 4 | FASLG | [356](https://www.ncbi.nlm.nih.gov/gene/356) | 31 | EGFR | [1956](https://www.ncbi.nlm.nih.gov/gene/1956) | 58 | BCL2L1 | [598](https://www.ncbi.nlm.nih.gov/gene/598) |
| 5 | CYP19A1 | [1588](https://www.ncbi.nlm.nih.gov/gene/1588) | 32 | ABCC1 | [4363](https://www.ncbi.nlm.nih.gov/gene/4363) | 59 | CCNA2 | [890](https://www.ncbi.nlm.nih.gov/gene/890) |
| 6 | KDR | [3791](https://www.ncbi.nlm.nih.gov/gene/3791) | 33 | DPP4 | [1803](https://www.ncbi.nlm.nih.gov/gene/1803) | 60 | TIMP1 | [7076](https://www.ncbi.nlm.nih.gov/gene/7076) |
| 7 | TP53 | [7157](https://www.ncbi.nlm.nih.gov/gene/7157) | 34 | IL1B | [3553](https://www.ncbi.nlm.nih.gov/gene/3553) | 61 | CASP8 | [841](https://www.ncbi.nlm.nih.gov/gene/841) |
| 8 | PTEN | [5728](https://www.ncbi.nlm.nih.gov/gene/5728) | 35 | SOD1 | [6647](https://www.ncbi.nlm.nih.gov/gene/6647) | 62 | AR | [367](https://www.ncbi.nlm.nih.gov/gene/367) |
| 9 | CYCS | [54205](https://www.ncbi.nlm.nih.gov/gene/54205) | 36 | CCND1 | [595](https://www.ncbi.nlm.nih.gov/gene/595) | 63 | PPARG | [5468](https://www.ncbi.nlm.nih.gov/gene/5468) |
| 10 | NOS2 | [4843](https://www.ncbi.nlm.nih.gov/gene/4843) | 37 | IL2 | [3558](https://www.ncbi.nlm.nih.gov/gene/3558) | 64 | BAX | [581](https://www.ncbi.nlm.nih.gov/gene/581) |
| 11 | TNF | [7124](https://www.ncbi.nlm.nih.gov/gene/7124) | 38 | ESR1 | [2099](https://www.ncbi.nlm.nih.gov/gene/2099) | 65 | GSK3B | [2932](https://www.ncbi.nlm.nih.gov/gene/2932) |
| 12 | RAF1 | [5894](https://www.ncbi.nlm.nih.gov/gene/5894) | 39 | CREB1 | [1385](https://www.ncbi.nlm.nih.gov/gene/1385) | 66 | CRP | [1401](https://www.ncbi.nlm.nih.gov/gene/1401) |
| 13 | CCNB1 | [891](https://www.ncbi.nlm.nih.gov/gene/891) | 40 | ERBB2 | [2064](https://www.ncbi.nlm.nih.gov/gene/2064) | 67 | HIF1A | [3091](https://www.ncbi.nlm.nih.gov/gene/3091) |
| 14 | EGF | [1950](https://www.ncbi.nlm.nih.gov/gene/1950) | 41 | CDK1 | [983](https://www.ncbi.nlm.nih.gov/gene/983) | 68 | CD40LG | [959](https://www.ncbi.nlm.nih.gov/gene/959) |
| 15 | IL1A | [3552](https://www.ncbi.nlm.nih.gov/gene/3552) | 42 | CDK4 | [1019](https://www.ncbi.nlm.nih.gov/gene/1019) | 69 | MMP1 | [4312](https://www.ncbi.nlm.nih.gov/gene/4312) |
| 16 | MMP3 | [4314](https://www.ncbi.nlm.nih.gov/gene/4314) | 43 | IGFBP3 | [3486](https://www.ncbi.nlm.nih.gov/gene/3486) | 70 | STAT1 | [6772](https://www.ncbi.nlm.nih.gov/gene/6772) |
| 17 | MET | [4233](https://www.ncbi.nlm.nih.gov/gene/4233) | 44 | IFNG | [3458](https://www.ncbi.nlm.nih.gov/gene/3458) | 71 | IL6 | [3569](https://www.ncbi.nlm.nih.gov/gene/3569) |
| 18 | CDKN1A | [1026](https://www.ncbi.nlm.nih.gov/gene/1026) | 45 | HSPA5 | [3309](https://www.ncbi.nlm.nih.gov/gene/3309) | 72 | CXCL8 | [3576](https://www.ncbi.nlm.nih.gov/gene/3576) |
| 19 | CAT | [847](https://www.ncbi.nlm.nih.gov/gene/847) | 46 | PTGS1 | [5742](https://www.ncbi.nlm.nih.gov/gene/5742) | 73 | SELE | [6401](https://www.ncbi.nlm.nih.gov/gene/6401) |
| 20 | FASN | [2194](https://www.ncbi.nlm.nih.gov/gene/2194) | 47 | IL4 | [3565](https://www.ncbi.nlm.nih.gov/gene/3565) | 74 | CDK2 | [1017](https://www.ncbi.nlm.nih.gov/gene/1017) |
| 21 | PCNA | [5111](https://www.ncbi.nlm.nih.gov/gene/5111) | 48 | CHEK1 | [1111](https://www.ncbi.nlm.nih.gov/gene/1111) | 75 | AHR | [196](https://www.ncbi.nlm.nih.gov/gene/196) |
| 22 | AKT1 | [207](https://www.ncbi.nlm.nih.gov/gene/207) | 49 | BCL2 | [596](https://www.ncbi.nlm.nih.gov/gene/596) | 76 | CASP3 | [836](https://www.ncbi.nlm.nih.gov/gene/836) |
| 23 | IL10 | [3586](https://www.ncbi.nlm.nih.gov/gene/3586) | 50 | HK2 | [3099](https://www.ncbi.nlm.nih.gov/gene/3099) | 77 | ODC1 | [4953](https://www.ncbi.nlm.nih.gov/gene/4953) |
| 24 | STAT3 | [6774](https://www.ncbi.nlm.nih.gov/gene/6774) | 51 | VEGFA | [7422](https://www.ncbi.nlm.nih.gov/gene/7422) | 78 | MMP9 | [4318](https://www.ncbi.nlm.nih.gov/gene/4318) |
| 25 | MAPK1 | [5594](https://www.ncbi.nlm.nih.gov/gene/5594) | 52 | TOP2A | [7153](https://www.ncbi.nlm.nih.gov/gene/7153) | 79 | TOP1 | [7150](https://www.ncbi.nlm.nih.gov/gene/7150) |
| 26 | PTGS2 | [5743](https://www.ncbi.nlm.nih.gov/gene/5743) | 53 | TGFB1 | [7040](https://www.ncbi.nlm.nih.gov/gene/7040) | 80 | ADIPOQ | [9370](https://www.ncbi.nlm.nih.gov/gene/9370) |
| 27 | BIRC5 | [332](https://www.ncbi.nlm.nih.gov/gene/332) | 54 | MAPK8 | [5599](https://www.ncbi.nlm.nih.gov/gene/5599) |  |  |  |

**Wang *et al*., Table 4**

**Table 4. The candidate ingredients of XFZYD for anti-tumour activity**

| Herbs | Type | Ingredients | MW | OB (%) | DL |
| --- | --- | --- | --- | --- | --- |
| *Angellica sinensis* (Oliv) Diels., *Rehmannia glutinosa* Libosch., *Prunus persica* (L.) Batsch, *Carthamus tinctorius* L., *Citrus aurantium* L., *Paeonia lactiflora* Pall., *Cyathula officinalis* Kuan | A1 | beta-sitosterol | 414.79 | 36.91 | 0.75 |
| *Angellica sinensis* (Oliv) Diels., *Carthamus tinctorius* L., *Paeonia lactiflora* Pall., *Bupleurum chinense* DC., | A2 | Stigmasterol | 412.77 | 43.83 | 0.76 |
| *Bupleurum chinense* DC., *Carthamus tinctorius* L., *Glycyrrhiza uralensis* Fisch., *Cyathula officinalis* Kuan | A3 | quercetin | 302.25 | 46.43 | 0.28 |
| *Carthamus tinctorius* L., *Bupleurum chinense* DC., *Glycyrrhiza uralensis* Fisch. | A4 | kaempferol | 286.25 | 41.88 | 0.24 |
| *Rehmannia glutinosa* Libosch., *Prunus persica* (L.) Batsch, | A5 | campesterol | 400.76 | 37.58 | 0.71 |
| *Paeonia lactiflora* Pall., *Carthamus tinctorius* L. | A6 | baicalein | 270.25 | 33.52 | 0.21 |
| *Carthamus tinctorius* L., *Platycodon grandiflorum* (Jacq.) | A7 | luteolin | 286.25 | 36.16 | 0.25 |
| *Citrus aurantium* L., *Glycyrrhiza uralensis* Fisch. | A8 | naringenin | 272.27 | 59.29 | 0.21 |
| *Bupleurum chinense* DC., *Glycyrrhiza uralensis* Fisch. | A9 | isorhamnetin | 316.28 | 49.6 | 0.31 |
| *Prunus persica* (L.) Batsch | TR1 | Sitosterol alpha1 | 426.8 | 43.28 | 0.78 |
|  | TR2 | 2,3-didehydro GA77 | 346.41 | 88.08 | 0.53 |
|  | TR3 | GA120 | 314.41 | 84.85 | 0.45 |
|  | TR4 | GA54 | 348.43 | 64.21 | 0.53 |
|  | TR5 | GA63 | 348.43 | 65.54 | 0.54 |
|  | TR6 | gibberellin 7 | 330.41 | 73.8 | 0.5 |
|  | TR7 | GA87 | 362.41 | 68.85 | 0.57 |
|  | TR8 | 3-O-p-coumaroylquinic acid | 338.34 | 37.63 | 0.29 |
|  | TR9 | hederagenin | 414.79 | 36.91 | 0.75 |
|  | TR10 | 2,3-didehydro GA70 | 330.41 | 63.29 | 0.5 |

**Table 4. The candidate ingredients of XFZYD for anti-tumour activity** (Continued)

| Herbs | Type | Ingredients | MW | OB (%) | DL |
| --- | --- | --- | --- | --- | --- |
| *Carthamus tinctorius* L. | HH1 | 4-[(E)-4-(3,5-dimethoxy-4-oxo-1-cyclohexa-2,5-dienylidene)but-2-enylidene]-2,6-dimethoxycyclohexa-2,5-dien-1-one | 356.4 | 48.47 | 0.36 |
|  | HH2 | lignan | 458.55 | 43.32 | 0.65 |
|  | HH3 | Pyrethrin II | 372.5 | 48.36 | 0.35 |
|  | HH4 | 6-Hydroxykaempferol | 302.25 | 62.13 | 0.27 |
|  | HH5 | qt_carthamone | 286.25 | 51.03 | 0.2 |
|  | HH6 | quercetagetin | 318.25 | 45.01 | 0.31 |
|  | HH7 | 7,8-dimethyl-1H-pyrimido[5,6-g]quinoxaline-2,4-dione | 242.26 | 45.75 | 0.19 |
|  | HH8 | beta-carotene | 536.96 | 37.18 | 0.58 |
| *Citrus aurantium* L. | ZQ1 | Marmin | 332.43 | 38.23 | 0.31 |
|  | ZQ2 | Hesperetin | 302.3 | 70.31 | 0.27 |
|  | ZQ3 | nobiletin | 402.43 | 61.67 | 0.52 |
| *Paeonia lactiflora* Pall. | CS1 | ellagic acid | 302.2 | 43.06 | 0.43 |
|  | CS2 | paeoniflorin | 480.51 | 53.87 | 0.79 |
|  | CS3 | (+)-catechin | 290.29 | 54.83 | 0.24 |
|  | CS4 | (2R,3R)-4-methoxyl-distylin | 318.3 | 59.98 | 0.3 |
| *Bupleurum chinense* DC. | CH1 | Linoleyl acetate | 308.56 | 42.1 | 0.2 |
|  | CH2 | 3,5,6,7-tetramethoxy-2-(3,4,5-trimethoxyphenyl)chromone | 432.46 | 31.97 | 0.59 |
|  | CH3 | Areapillin | 360.34 | 48.96 | 0.41 |
|  | CH4 | Cubebin | 356.4 | 57.13 | 0.64 |
|  | CH5 | (+)-Anomalin | 426.5 | 46.06 | 0.66 |
|  | CH6 | petunidin | 317.29 | 30.05 | 0.31 |
| *Glycyrrhiza uralensis* Fisch. | GC1 | Inermine | 284.28 | 75.18 | 0.54 |
|  | GC2 | DFV | 256.27 | 32.76 | 0.18 |
|  | GC3 | Glycyrol | 366.39 | 90.78 | 0.67 |
|  | GC4 | Jaranol | 314.31 | 50.83 | 0.29 |
|  | GC5 | Medicarpin | 270.3 | 49.22 | 0.34 |
|  | GC6 | Lupiwighteone | 338.38 | 51.64 | 0.37 |

**Table 4. The candidate ingredients of XFZYD for anti-tumour activity** (Continued)

| Herbs | Type | Ingredients | MW | OB (%) | DL |
| --- | --- | --- | --- | --- | --- |
| *Glycyrrhiza uralensis* Fisch. | GC7 | 7-Methoxy-2-methyl isoflavone | 266.31 | 42.56 | 0.2 |
|  | GC8 | formononetin | 268.28 | 69.67 | 0.21 |
|  | GC9 | Calycosin | 284.28 | 47.75 | 0.24 |
|  | GC10 | (2S)-2-[4-hydroxy-3-(3-methylbut-2-enyl)phenyl]-8,8-dimethyl-2,3-dihydropyrano[2,3-f]chromen-4-one | 390.51 | 31.79 | 0.72 |
|  | GC11 | euchrenone | 406.56 | 30.29 | 0.57 |
|  | GC12 | glyasperin B | 370.43 | 65.22 | 0.44 |
|  | GC13 | glyasperin F | 354.38 | 75.84 | 0.54 |
|  | GC14 | Glyasperin C | 356.45 | 45.56 | 0.4 |
|  | GC15 | Isotrifoliol | 298.26 | 31.94 | 0.42 |
|  | GC16 | (E)-1-(2,4-dihydroxyphenyl)-3-(2,2-dimethylchromen-6-yl)prop-2-en-1-one | 322.38 | 39.62 | 0.35 |
|  | GC17 | kanzonols W | 336.36 | 50.48 | 0.52 |
|  | GC18 | (2S)-6-(2,4-dihydroxyphenyl)-2-(2-hydroxypropan-2-yl)-4-methoxy-2,3-dihydrofuro[3,2-g]chromen-7-one | 384.41 | 60.25 | 0.63 |
|  | GC19 | Semilicoisoflavone B | 352.36 | 48.78 | 0.55 |
|  | GC20 | Glepidotin A | 338.38 | 44.72 | 0.35 |
|  | GC21 | Glepidotin B | 340.4 | 64.46 | 0.34 |
|  | GC22 | Phaseolinisoflavan | 324.4 | 32.01 | 0.45 |
|  | GC23 | Glypallichalcone | 284.33 | 61.6 | 0.19 |
|  | GC24 | 8-(6-hydroxy-2-benzofuranyl)-2,2-dimethyl-5-chromenol | 308.35 | 58.44 | 0.38 |
|  | GC25 | Licochalcone B | 286.3 | 76.76 | 0.19 |
|  | GC26 | licochalcone G | 354.43 | 49.25 | 0.32 |

**Table 4. The candidate ingredients of XFZYD for anti-tumour activity** (Continued)

| Herbs | Type | Ingredients | MW | OB (%) | DL |
| --- | --- | --- | --- | --- | --- |
| *Glycyrrhiza uralensis* Fisch. | GC27 | 3-(2,4-dihydroxyphenyl)-8-(1,1-dimethylprop-2-enyl)-7-hydroxy-5-methoxy-coumarin | 368.41 | 59.62 | 0.43 |
|  | GC28 | Licoricone | 382.44 | 63.58 | 0.47 |
|  | GC29 | Gancaonin A | 352.41 | 51.08 | 0.4 |
|  | GC30 | Gancaonin B | 368.41 | 48.79 | 0.45 |
|  | GC31 | 3-(3,4-dihydroxyphenyl)-5,7-dihydroxy-8-(3-methylbut-2-enyl)chromone | 354.38 | 66.37 | 0.41 |
|  | GC32 | 5,7-dihydroxy-3-(4-methoxyphenyl)-8-(3-methylbut-2-enyl)chromone | 352.41 | 30.49 | 0.41 |
|  | GC33 | 2-(3,4-dihydroxyphenyl)-5,7-dihydroxy-6-(3-methylbut-2-enyl)chromone | 354.38 | 44.15 | 0.41 |
|  | GC34 | Glycyrin | 382.44 | 52.61 | 0.47 |
|  | GC35 | Licocoumarone | 340.4 | 33.21 | 0.36 |
|  | GC36 | Licoisoflavone | 354.38 | 41.61 | 0.42 |
|  | GC37 | Licoisoflavone B | 352.36 | 38.93 | 0.55 |
|  | GC38 | licoisoflavanone | 354.38 | 52.47 | 0.54 |
|  | GC39 | shinpterocarpin | 322.38 | 80.3 | 0.73 |
|  | GC40 | (E)-3-[3,4-dihydroxy-5-(3-methylbut-2-enyl)phenyl]-1-(2,4-dihydroxyphenyl)prop-2-en-1-one | 340.4 | 46.27 | 0.31 |
|  | GC41 | liquiritin | 418.43 | 65.69 | 0.74 |
|  | GC42 | licopyranocoumarin | 384.41 | 80.36 | 0.65 |
|  | GC43 | Glyzaglabrin | 298.26 | 61.07 | 0.35 |
|  | GC44 | Glabridin | 324.4 | 53.25 | 0.47 |
|  | GC45 | Glabrene | 322.38 | 46.27 | 0.44 |

**Table 4. The candidate ingredients of XFZYD for anti-tumour activity** (Continued)

| Herbs | Type | Ingredients | MW | OB (%) | DL |
| --- | --- | --- | --- | --- | --- |
| *Glycyrrhiza uralensis* Fisch. | GC46 | 1,3-dihydroxy-9-methoxy-6-benzofurano[3,2-c]chromenone | 298.26 | 48.14 | 0.43 |
|  | GC47 | Eurycarpin A | 338.38 | 43.28 | 0.37 |
|  | GC48 | (-)-Medicocarpin | 432.46 | 40.99 | 0.95 |
|  | GC49 | Sigmoidin-B | 356.4 | 34.88 | 0.41 |
|  | GC50 | (2R)-7-hydroxy-2-(4-hydroxyphenyl) chroman-4-one | 256.27 | 71.12 | 0.18 |
|  | GC51 | Isoglycyrol | 366.39 | 44.7 | 0.84 |
|  | GC52 | Isolicoflavonol | 354.38 | 45.17 | 0.42 |
|  | GC53 | HMO | 268.28 | 38.37 | 0.21 |
|  | GC54 | 1-Methoxyphaseollidin | 354.43 | 69.98 | 0.64 |
|  | GC55 | 3'-Hydroxy-4'-O-Methylglabridin | 354.43 | 43.71 | 0.57 |
|  | GC56 | licochalcone a | 338.43 | 40.79 | 0.29 |
|  | GC57 | 3'-Methoxyglabridin | 354.43 | 46.16 | 0.57 |
|  | GC58 | 2-[(3R)-8,8-dimethyl-3,4-dihydro-2H-pyrano[6,5-f]chromen-3-yl]-5-methoxyphenol | 338.43 | 36.21 | 0.52 |
|  | GC59 | Inflacoumarin A | 322.38 | 39.71 | 0.33 |
|  | GC60 | Kanzonol F | 420.54 | 32.47 | 0.89 |
|  | GC61 | 6-prenylated eriodictyol | 356.4 | 39.22 | 0.41 |
|  | GC62 | 7,2',4'-trihydroxy－5-methoxy-3－arylcoumarin | 300.28 | 83.71 | 0.27 |
|  | GC63 | 7-Acetoxy-2-methylisoflavone | 294.32 | 38.92 | 0.26 |
|  | GC64 | 8-prenylated eriodictyol | 356.4 | 53.79 | 0.4 |
|  | GC65 | Vestitol | 272.32 | 74.66 | 0.21 |
|  | GC66 | Gancaonin G | 352.41 | 60.44 | 0.39 |
|  | GC67 | Gancaonin H | 420.49 | 50.1 | 0.78 |
|  | GC68 | Licoagrocarpin | 338.43 | 58.81 | 0.58 |
|  | GC69 | Glyasperins M | 368.41 | 72.67 | 0.59 |

**Table 4. The candidate ingredients of XFZYD for anti-tumour activity** (Continued)

| Herbs | Type | Ingredients | MW | OB (%) | DL |
| --- | --- | --- | --- | --- | --- |
| *Glycyrrhiza uralensis* Fisch. | GC70 | Glycyrrhiza flavonol A | 370.38 | 41.28 | 0.6 |
|  | GC71 | Licoagroisoflavone | 336.36 | 57.28 | 0.49 |
|  | GC72 | Odoratin | 314.31 | 49.95 | 0.3 |
|  | GC73 | Phaseol | 336.36 | 78.77 | 0.58 |
|  | GC74 | Xambioona | 388.49 | 54.85 | 0.87 |
|  | GC75 | dehydroglyasperins C | 340.4 | 53.82 | 0.37 |
|  | GC76 | Quercetin der. | 330.31 | 46.45 | 0.33 |
|  | GC77 | (2S)-7-hydroxy-2-(4-hydroxyphenyl)-8-(3-methylbut-2-enyl)chroman-4-one | 324.4 | 36.57 | 0.32 |
|  | GC78 | 1,3-dihydroxy-8,9-dimethoxy-6-benzofurano[3,2-c]chromenone | 328.29 | 62.9 | 0.53 |
|  | GC79 | Glabrone | 336.36 | 52.51 | 0.5 |
|  | GC80 | Glabranin | 324.4 | 52.9 | 0.31 |
| Platycodon grandiflorum (Jacq.) | JG1 | cis-Dihydroquercetin | 304.27 | 66.44 | 0.27 |
|  | JG2 | acacetin | 284.28 | 34.97 | 0.24 |
| Ligusticum chuanxiong Hort. | CX1 | Mandenol | 308.56 | 42 | 0.19 |
|  | CX2 | Myricanone | 356.45 | 40.6 | 0.51 |
|  | CX3 | Perlolyrine | 264.3 | 65.95 | 0.27 |
|  | CX4 | wallichilide | 412.57 | 42.31 | 0.71 |
|  | CX5 | FA | 441.45 | 68.96 | 0.71 |
| Cyathula officinalis Kuan | CNX1 | Betavulgarin | 312.29 | 68.75 | 0.39 |
