# Supplementary material for: Analysis of the Anti-Tumour Effect of Xuefu Zhuyu Decoction Based on Network Pharmacology and Experimental Verification in Drosophila
Source: Front Pharmacol. 2022 Jul 12;13:922457. doi: 10.3389/fphar.2022.922457 (PMC9315317; doi:10.3389/fphar.2022.922457)
Supplement: Supplementary file 5 [file Image4.PDF]

Analysis of the anti-tumour effect of Xuefu Zhuyu decoction based on network pharmacology and experimental verification in *Drosophila*

Sitong Wang[1,2†], Chenxi Wu[1†], Yinghong Li[1], Bin Ye[1], Shuai Wang[1], Guowang Li[1], Jiawei Wu[1], Shengnan Liu[1], Menglong Zhang[1], Yongsen Jia[1], Huijuan Cao[1], Chunhua Jiang[1*] and Fanwu Wu[1*]

[1] Hebei Key Laboratory of Integrated Traditional Chinese and Western Medicine for Diabetes and Its Complications, College of Traditional Chinese Medicine, North China University of Science and Technology, 21 Bohai Road, Tangshan 063210, China

[2] School of Traditional Chinese Medicine, Beijing University of Chinese Medicine, Beijing 100029, China

[†] These authors have contributed equally to this work and share first authorship.

[*] **Correspondence:**
Chunhua Jiang, Fanwu Wu
jiangchunhua@ncst.edu.cn, ldwfw@sina.com

Supplementary Figures

Wang *et al.*, Figure. 1

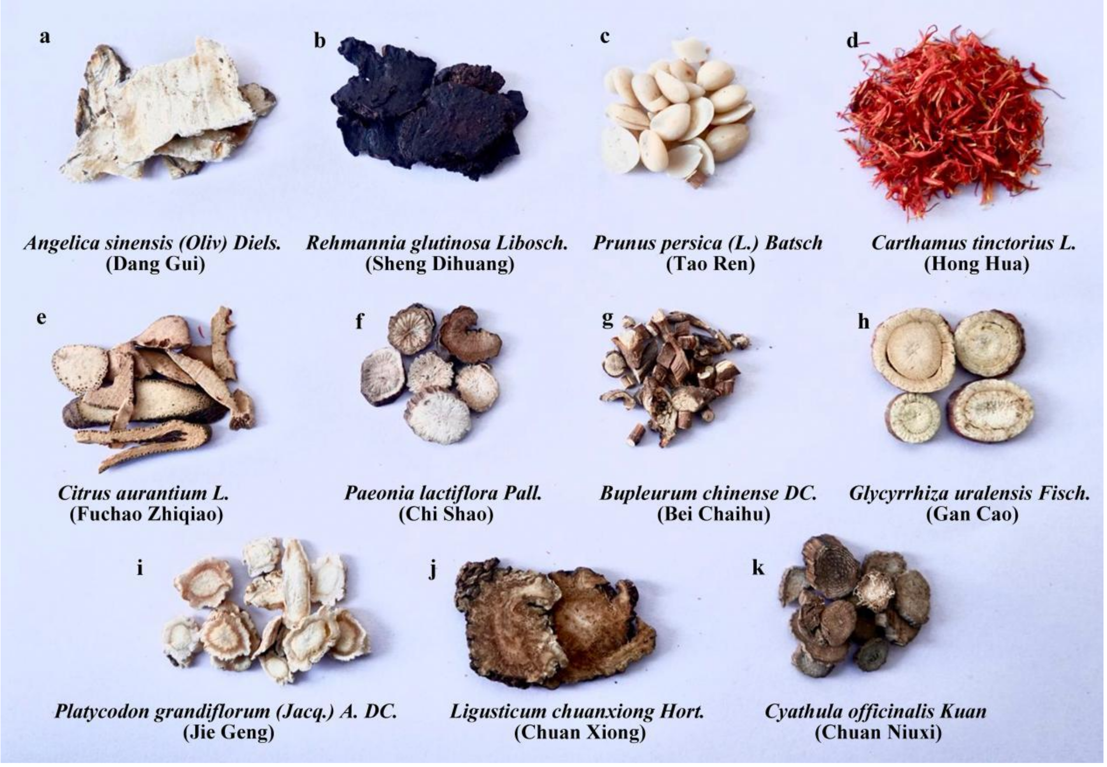

Figure 1. Composition of XFZYD.

(**a**–**k**) Photographs showing the composition of XFZYD: *Angelica sinensis* (Dang Gui, **a**), *Rehmannia glutinosa* (Sheng Dihuang, **b**), *Prunus persica* (Tao Ren, **c**), *Carthamus tinctorius* (Hong Hua, **d**), *Citrus aurantium* (Fuchao Zhiqiao, **e**), *Paeonia lactiflora* (Chi Shao, **f**), *Bupleurum chinense* (Bei Chaihu, **g**), *Glycyrrhiza uralensis* (Gan Cao, **h**), *Platycodon grandiflorum* (Jie Geng, **i**), *Ligusticum chuanxiong* (Chuan Xiong, **j**), and *Cyathula officinalis* (Chuan Niuxi, **k**).

Wang *et al*., Figure 2

♀*yw ey*-Flp; *tub*-GAL80 FRT40A; *act*>y^+>GAL4 *UAS*-GFP × ♂*w*; lgl^4 FRT40A *UAS*-Ras^{V12} /*Cyo*

↓

yw ey-Flp/+; *tub*-GAL80 FRT40A/lgl^4 FRT40A *UAS*-Ras^{V12}; *act*>y^+>GAL4 *UAS*-GFP/+

(Ras^{V12} /$lgl^{-/-}$)

Figure 2. A flow chart for establishing the Ras^{V12}/$lgl^{-/-}$ model.
Healthy unmated female flies with genotype *yw ey*-Flp; *tub*-GAL80 FRT40A; *act*>y^+>GAL4 *UAS*-GFP were crossed to male flies with genotype *w; lgl*^{4} FRT40A *UAS*-Ras^{V12}/*Cyo*. These fruit flies mated and laid eggs in one tube containing normal food. When the offspring reached the 3rd instar larval stage, larvae with the *yw ey*-Flp/+; *tub*-GAL80 FRT40A/lgl^4 FRT40A *UAS*-Ras^{V12}; *act*>y^+>GAL4 *UAS*-GFP/+ (Ras^{V12}/$lgl^{-/-}$) genotype were collected and dissected.

Wang *et al.*, Figure 3

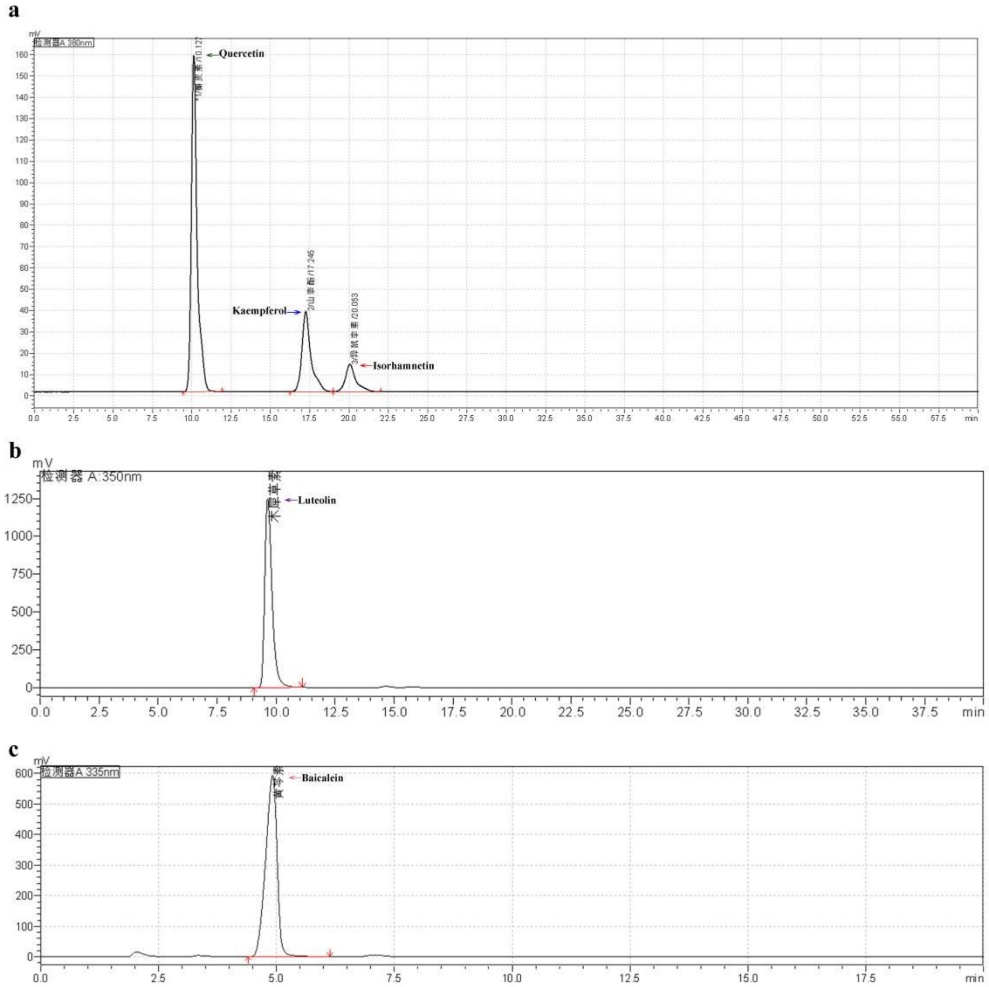

Figure 3. The HPLC chromatogram of standard controls.

Standards for quercetin (**a**), kaempferol (**a**), isorhamnetin (**a**), luteolin (**b**), and baicalein (**c**) in XFZYD. The tested peaks are indicated with green, blue, red, purple, and pink arrows, respectively.
